# The phosphatase Glc7 controls the eisosomal response to starvation via post-translational modification of Pil1

**DOI:** 10.1242/jcs.260505

**Published:** 2023-07-24

**Authors:** Katherine M. Paine, Kamilla M. E. Laidlaw, Gareth J. O. Evans, Chris MacDonald

**Affiliations:** ^1^ York Biomedical Research Institute. University of York, York YO10 5DD, UK; ^2^ Department of Biology, University of York, York YO10 5DD, UK

**Keywords:** Eisosomes, Nutrient regulation, Phosphorylation, Plasma membrane, Yeast

## Abstract

The yeast (*Saccharomyces cerevisiae*) plasma membrane (PM) is organised into specific subdomains that regulate surface membrane proteins. Surface transporters actively uptake nutrients in particular regions of the PM where they are also susceptible to substrate-induced endocytosis. However, transporters also diffuse into distinct subdomains termed eisosomes, where they are protected from endocytosis. Although most nutrient transporter populations are downregulated in the vacuole following glucose starvation, a small pool is retained in eisosomes to provide efficient recovery from starvation. We find the core eisosome subunit Pil1, a Bin, Amphiphysin and Rvs (BAR) domain protein required for eisosome biogenesis, is phosphorylated primarily by the kinase Pkh2. In response to acute glucose starvation, Pil1 is rapidly dephosphorylated. Enzyme localisation and activity screens suggest that the phosphatase Glc7 is the primary enzyme responsible for Pil1 dephosphorylation. Defects in Pil1 phosphorylation, achieved by depletion of *GLC7* or expression of phospho-ablative or phospho-mimetic mutants, correlate with reduced retention of transporters in eisosomes and inefficient starvation recovery. We propose that precise post-translational control of Pil1 modulates nutrient transporter retention within eisosomes, depending on extracellular nutrient levels, to maximise recovery following starvation.

## Teaser

**Summary:** Glc7-mediated dephosphorylation of the yeast BAR domain protein Pil1 modulates plasma membrane subdomains to promote transporter retention and recovery potential.

## INTRODUCTION

The plasma membrane (PM) of eukaryotic cells is organised into distinct domains of specific lipids and proteins (Kraft, 2013). In the budding yeast *Saccharomyces cerevisiae*, distinct spatiotemporal localisation patterns have been observed for different proteins (Berchtold and Walther, 2009; Grossmann et al., 2007; Heinisch et al., 2010; Murley et al., 2017; Spira et al., 2012). Original localisation studies distinguished between the hexose transporter Hxt1, which is uniformly dispersed across the surface, and other proteins, such as Pma1 and Can1, which are found in discrete, non-overlapping regions (Malínská et al., 2003). The punctate subdomains occupied by the arginine transporter Can1, originally termed the membrane compartment of Can1 (MCC) and later denoted as eisosomes (Walther et al., 2006), have also been shown to house many other nutrient transporters (Babst, 2019). Eisosomes have been identified in other fungal species, such as *Aspergillus nidulans* and *Ashbya gossypii* (Seger et al., 2011; Vangelatos et al., 2010), as well as various species of lichens and algae (Lee et al., 2015).

Eisosomes are furrow-like PM structures enriched in sterols and sphingolipids (Grossmann et al., 2007; Malínská et al., 2003; StráDalová et al., 2009). Eisosome formation occurs *de novo*, and although once formed these structures are relatively immobile, core proteins remain dynamic (Moreira et al., 2009; Olivera-Couto et al., 2015; Walther et al., 2007). Many proteins localise to eisosomes, such as core structural proteins, post-translational modifiers, tetraspan membrane proteins and uncharacterised factors (Foderaro et al., 2017). For example, the tetraspanner Nce102, which functions as a sphingolipid sensor and promotes membrane curvature (Fröhlich et al., 2009; Haase et al., 2022 preprint; Vaskovicova et al., 2020; Zahumenský et al., 2022), and Seg1, a stability factor that operates upstream of eisosome formation (Moreira et al., 2012; Seger et al., 2011). A screen for phosphatidylinositol (4,5)-bisphosphate [PI(4,5)P_2_] regulators has revealed that the eisosome factors Slm1 and Slm2 bind lipids, are required for proper eisosomal organisation, and integrate with TORC2 signalling and lipid synthesis (Audhya et al., 2004; Berchtold et al., 2012; Fadri et al., 2005; Kamble et al., 2011; Nagaya et al., 2002; Riggi et al., 2018). The Bin, Amphiphysin and Rvs (BAR) domain proteins Pil1 and Lsp1 are required for organising lipids during the sculpting of eisosomes (Moreira et al., 2009; Walther et al., 2006; Zhao et al., 2013; Ziółkowska et al., 2011). Pil1 and Lsp1 freely diffuse in the cytoplasm but almost exclusively localise to eisosomes at steady state (Olivera-Couto et al., 2015). Under stress conditions, Lsp1 can at least partially complement loss of Pil1 (Vesela et al., 2023). Pil1 and Lsp1 are phosphorylated by kinases Slt2, Pkh1 and Pkh2, and the consequences of Pil1 phosphorylation on eisosome biogenesis have been characterised previously (Mascaraque et al., 2013; Walther et al., 2007; Zhang et al., 2004).

Yeast cells uptake nutrients from their external environment through specific transporters that localise to the PM (Jack et al., 2000; Léon and Teis, 2018). Regulation of these transporters at the PM allows for nutrient acquisition to be tightly controlled in response to cellular requirements. Active transporters localised to the PM, like Fur4, Can1 and Mup1, undergo conformational changes in response to nutrients and are more efficiently serviced by the endocytic machinery (Gournas et al., 2017; Guiney et al., 2016; Keener and Babst, 2013). Nutrient transporter ubiquitylation is the signal for trafficking through the multivesicular body (MVB) pathway, wherein ubiquitylated proteins are recognised and packaged into intralumenal vesicles of the MVB by the endosomal sorting complex required for transport (ESCRT) apparatus (Migliano et al., 2022). Upon MVB–vacuole fusion, intraluminal vesicles containing surface proteins are deposited in the degradative environment of the vacuolar lumen. These feedback mechanisms allow for transporter degradation to avoid excessive nutrient uptake, which can be detrimental (Kaur and Bachhawat, 2007; Séron et al., 1999; Watanabe et al., 2014). These trafficking events are coordinated in response to nutritional cues, for example, in response to nitrogen starvation, surface proteins are degraded more readily owing to the elevation in vacuolar sorting triggered via Rsp5 and its adaptors (Ivashov et al., 2020; MacGurn et al., 2011; Müller et al., 2015). Rsp5-mediated degradation is also upregulated in response to growth past log-phase, when niacin becomes limited (MacDonald et al., 2015). Surface proteins are also degraded faster and recycled less efficiently in response to leucine starvation (Jones et al., 2012; MacDonald and Piper, 2017). A similar dual control of trafficking pathways in response to glucose starvation, which triggers surface protein degradation (Lang et al., 2014), occurs through an increase in AP180-mediated endocytosis and a decrease in Gpa1–phosphoinositide 3-kinase-mediated recycling (Laidlaw et al., 2021, 2022b).

The tendency of nutrient transporters like Can1, Fur4 and Mup1 to also localise to eisosomes has led to various investigations into what regulatory control is provided within these subdomains (Busto et al., 2018; Gournas et al., 2018a,b; Moharir et al., 2018). Although activity of these transporters might vary when localised to eisosomes, the consensus view that eisosomes provide protection from ubiquitin-mediated endocytosis has been demonstrated for all (Athanasopoulos et al., 2019). These surface cargoes have also been used in the context of stress condition experiments. Following stress, nutrient transporter populations are not entirely degraded, with a small proportion of the cellular pool being sequestrated in eisosomes. Unlike the response to substrates, where transporters like Can1, Fur4 and Mup1 move from eisosomes and undergo endocytosis, starvation conditions, such as a poor nitrogen source or growth to stationary phase, results in increased transporter concentration in eisosomes (Gournas et al., 2018b; Moharir et al., 2018). Stress conditions trigger restructuring of eisosomes, with changes in PM tension and deepening of these structures, to better retain this reserve pool of nutrient transporters (Appadurai et al., 2020; Moharir et al., 2018; Riggi et al., 2018). Furthermore, there is a physiological benefit to harbouring these nutrient transporters in eisosomes – to allow efficient recovery following a return to replete conditions. For example, the uracil transporter Fur4, which is required at the surface for efficient growth in limited uracil conditions, contributes to efficient recovery following glucose starvation, in an uracil-dependent manner (Laidlaw et al., 2021; Paine et al., 2021). The retention of nutrient transporters in response to stress is not well understood at a mechanistic level. As mentioned, phosphorylation of the core factor Pil1 by Pkh kinases is an important regulatory step in eisosome biogenesis (Karotki et al., 2011; Luo et al., 2008; Walther et al., 2007). We find that under basal conditions, only Pkh2 predominantly localises to eisosomes and is largely responsible for phosphorylating Pil1.

As Pil1 is dephosphorylated in response to glucose starvation (Laidlaw et al., 2021), screening for responsible enzymes reveals the PP1 phosphatase Glc7 is a critical enzyme that regulates Pil1 dephosphorylation, nutrient transporter homeostasis and recovery from starvation. These effects are phenocopied upon mutation of Pil1 phospho-sites, suggesting that the phosphorylation status of Pil1 modulates eisosomes, not only during their biogenesis, but to retain nutrient transporters at the surface for recovery following starvation.

## RESULTS

### Pkh2 is the predominant kinase that phosphorylates Pil1

**Fig. 1.**
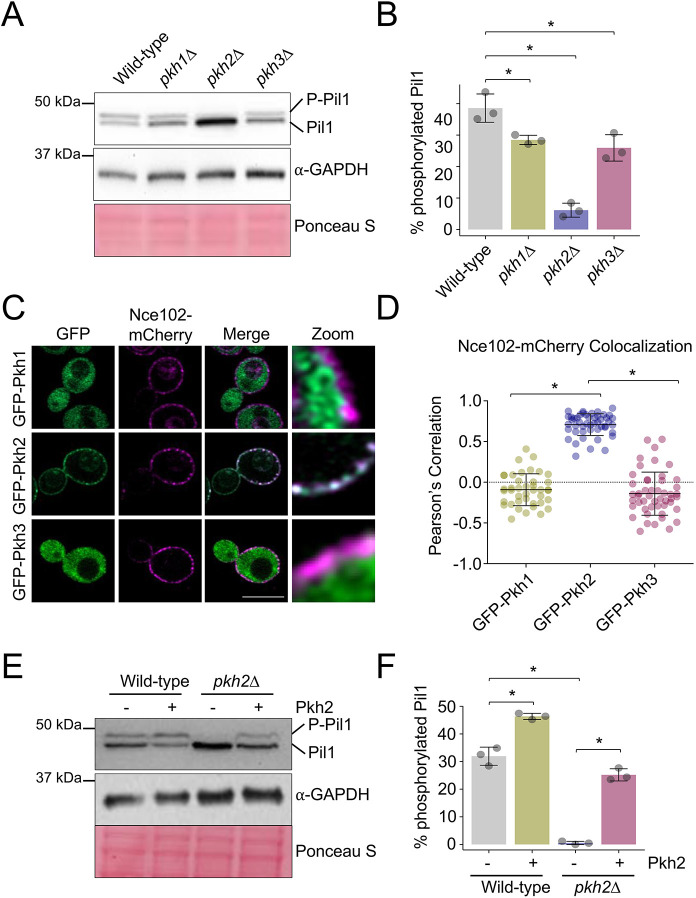
**Pkh2 predominately regulates phosphorylation of Pil1.** (A) Whole-cell lysates of wild-type, *pkh1*Δ, *pkh2*Δ and *pkh3*Δ cells were analysed by immunoblotting using anti-Pil1 and anti-GAPDH antibodies, including Ponceau S-stained membrane. P-Pil1, phosphorylated Pil1. (B) The percentage phosphorylated Pil1 from each yeast strain was quantified (*n*=3). (C) Cells co-expressing Nce102–mCherry and indicated GFP tagged Pkh kinases were grown to mid-log phase and imaged using confocal microscopy (Airyscan 2). Scale bar: 5 µm. (D) The Pearson's correlation coefficient was measured between Nce102–mCherry and the respective GFP-tagged kinases (*n*>40). (E) Wild-type and *pkh2*Δ cells were transformed with either an empty vector control (-) or a 2 µ Pkh2 over-expression plasmid (+). Whole-cell lysates were generated from transformants and Pil1 phosphorylation assessed by immunoblot. Levels of GAPDH and Ponceau S are shown as loading controls. (F) The percentage of phosphorylated Pil1 was quantified (*n*=3) and shown (right). Quantitative results are mean±s.d. *P<0.05 (unpaired *t*-test).

It has been previously shown that Pil1 phosphorylation is ablated in a double *pkh1^ts^ pkh2*Δ mutant (Luo et al., 2008; Walther et al., 2007). To determine the contribution of Pkh1 and Pkh2 to the phosphorylation of Pil1, we assessed individual deletion mutants. As Pkh3 was identified as a multicopy suppressor of *pkh^ts^pkh2* mutants (Inagaki et al., 1999), but has not been tested for a role in Pil1 phosphorylation, we included *pkh3*Δ mutants in this analysis. Pil1 phosphorylation status affects its migration during electrophoresis and can be visualised by immunoblotting (Walther et al., 2007). Pil1 phosphorylation was assessed in all three *pkh* mutants, which revealed that *pkh2*Δ mutants were most defective and that *pkh1*Δ and *pkh3*Δ cells have only a small but significant defect (Fig. 1A,B). We next performed localisation studies for these kinases. Although previous studies have demonstrated that over-expression of Pkh1 and Pkh2 with the galactose inducible promoter *GAL1* is required for sufficient levels to visualise localisation of these proteins (Roelants et al., 2002; Walther et al., 2007), we avoided this glucose-repression strategy, due to its effect on eisosome biology (Laidlaw et al., 2021). GFP-tagged kinases were instead over-expressed from the constitutive *NOP1* promoter (Weill et al., 2018) in cells co-expressing the eisosomal marker Nce102 tagged with mCherry. Only GFP–Pkh2 predominantly colocalised with Nce102–mCherry (Fig. 1C,D). This small apparent contribution of Pkh1 and Pkh3 to Pil1 phosphorylation observed by immunoblot might be explained by the fact that although most cells do not show an eisosome localisation for Pkh1 or Pkh3, a small number of cells do (Fig. S1A,B). In further support of Pkh2 being a regulator of Pil1 phosphorylation, overexpressing Pkh2 led to a significant increase in Pil1 phosphorylation, observed in both wild-type and *pkh2*Δ cells (Fig. 1E,F). These data demonstrate that Pkh2 is the primary Pkh family member responsible for Pil1 phosphorylation, but that Pkh1 and Pkh3 also exhibit subsidiary roles.

**Fig. 2.**
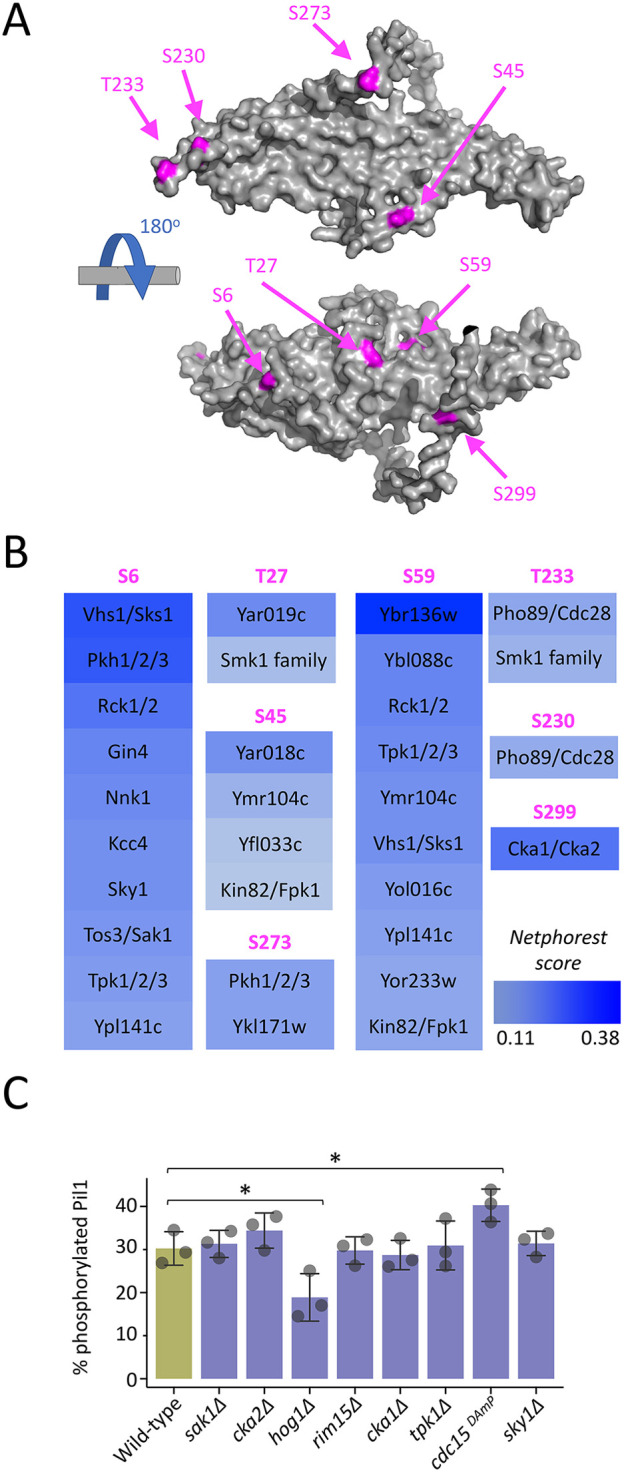
**Bioinformatic screen for additional kinases that service Pil1.** (A) Alphafold structural model of Pil1 residues 1–307 (grey) shown with eight previously verified phosphorylation sites indicated (magenta). (B) The Pil1 protein sequence was surveyed using NetPhorest searching against a reference kinase database for *Saccharomyces cerevisiae*. Kinases that scored above threshold (0.1) are presented as a heat map (blue) with the indicated potential phosphorylated residue (magenta). (C) Whole-cell lysates of wild-type and kinase mutant cells were generated and percentage Pil1 phosphorylation assessed by immunoblot and presented as a histogram (*n*=3). Results are mean±s.d. *P<0.05 (unpaired *t*-test).

Phosphorylated peptides of Pil1 have previously been identified by mass spectrometry (Albuquerque et al., 2008; Luo et al., 2008; Swaney et al., 2013; Walther et al., 2007), suggesting multiple levels of potential phospho-regulation. Experimental work has determined several key phospho-sites (Luo et al., 2008; Walther et al., 2007), which map to distinct regions of Pil1 (Fig. 2A). To ascertain whether any additional kinases beyond the Pkh family were responsible for Pil1 phosphorylation, NetPhorest analysis (Horn et al., 2014) was used to predict potential kinases for known Pil1 phospho-sites (Fig. 2B). Mutants of any high scoring kinases (Table S1) were tested for a role in phosphorylating Pil1 by immunoblotting (Fig. S2). Only eight mutants showed any indication of a potential role, which was followed up quantitatively. This revealed *hog1*Δ cells, lacking the yeast Hog1, a homologue of the mammalian p38 MAPKs (Han et al., 1994), had reduced levels of Pil1 phosphorylation. Also, depletion mutants with reduced levels of the Hippo-like kinase Cdc15 (Rock et al., 2013; Steensma et al., 1987), by virtue of a DAmP cassette (Breslow et al., 2008), showed higher levels of Pil1 phosphorylation (Fig. 2C). This additional analysis again supports the notion that Pkh2 is the primary enzyme responsible for Pil1 phosphorylation, but that phosphorylation via other kinases might also have an impact, potentially through indirect mechanisms, such as transcriptional or stress-induced control.

### Pil1 is dephopshorylated in response to glucose starvation

**Fig. 3.**
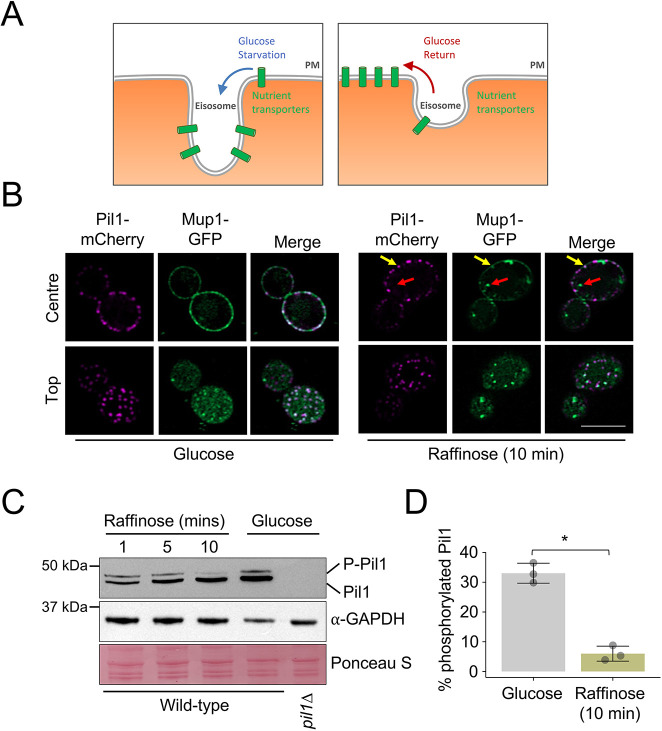
**Pil1 is dephosphorylated in response to glucose starvation.** (A) Schematic showing the increased diffusion of nutrient transporters into eisosomes in response to glucose starvation, and their potential exit to aid recovery in replete conditions. (B) Cells co-expressing Pil1–mCherry and Mup1–GFP were imaged using confocal microscopy (Airyscan 2) with a centre and top focus under glucose conditions and following 10 min of exchange with raffinose medium. Mup1 localised to endosomes (red arrow) and Pil1 marked eisosomes (yellow arrow) after raffinose treatment are indicated. Scale bar: 5 µm. (C) Wild-type cells exposed to raffinose medium for 1, 5 and 10 min prior to lysate generation were immunoblotted using α-Pil1 antibodies and compared to wild-type and *pil1*Δ cells grown in glucose-replete conditions. GAPDH blot and Ponceau S-stained membrane is included as a loading control. P-Pil1, phosphorylated Pil1. (D) Percentage of phosphorylated Pil1 was generated for WT versus 10 min of raffinose treatment for wild-type cells was quantified (*n*=3). Results are mean±s.d. *P<0.05 (unpaired *t*-test).

In response to acute glucose starvation, nutrient transporters localise to eisosomes (Laidlaw et al., 2021) and are hypothesised to relocate to PM regions for nutrient uptake upon return to replete conditions (Fig. 3A). For glucose starvation, a medium lacking glucose but containing the trisaccharide raffinose, which cannot be quickly metabolised (de la Fuente and Sols, 1962), is used. Upon raffinose exchange, nutrient transporters such as Mup1 are primarily downregulated; however, a small pool also concentrates within eisosomes (Laidlaw et al., 2021). This is best visualised by comparing puncta of Mup1–GFP in eisosomes marked by Pil1–mCherry in relation to Mup1, which is also diffusely localised to other PM regions, as seen in images taken as top-focussed confocal slices (Fig. 3B). During the initial period of glucose starvation when nutrient transporters accumulate in eisosomes, we observed rapid Pil1 dephosphorylation (Fig. 3C,D). We also observed significant glucose-induced dephosphorylation in *pkh1*Δ, moving to levels similar to *pkh2*Δ in basal conditions, again suggesting that Pkh1 is not a key regulator of Pil1 (Fig. S3A,B).

**Fig. 4.**
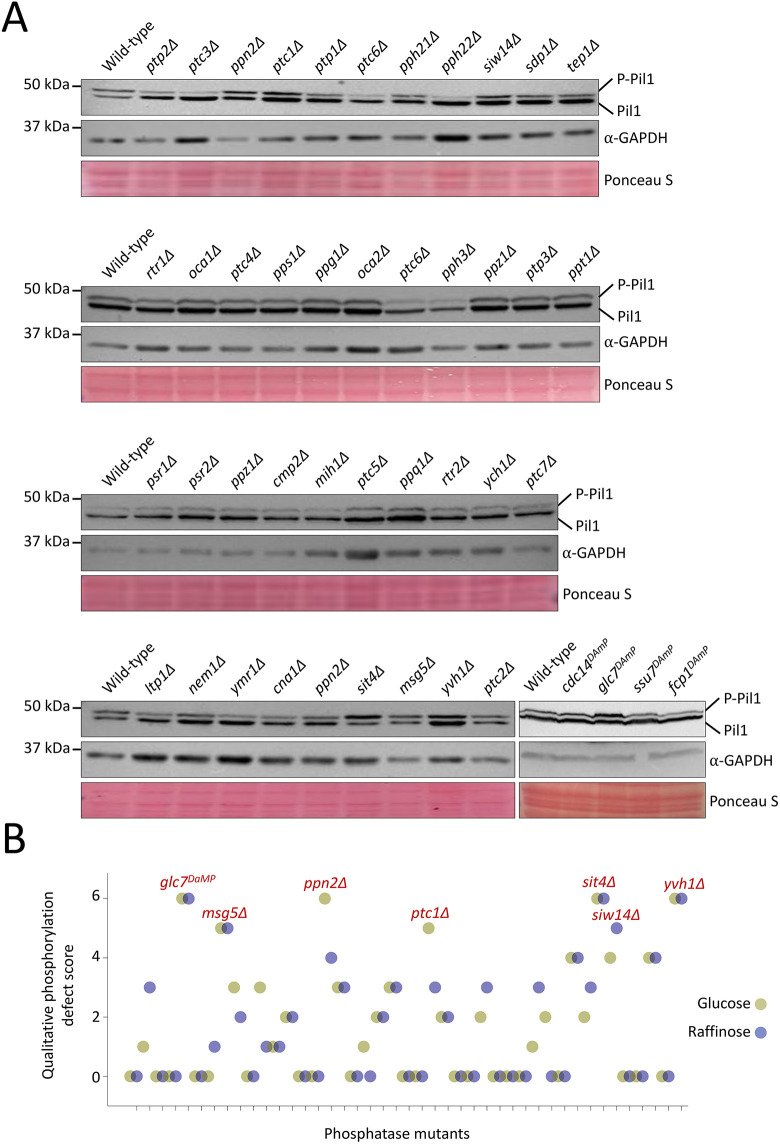
**Primary activity screen for regulators of Pil1 dephosphorylation.** (A) Wild-type cells and indicated phosphatase mutants were grown to log phase in glucose-replete conditions prior to lysate generation and immunoblotting with anti-Pil1 and anti-GAPDH antibodies. Representative blots of three experiments for at least one mutant are shown, alongside a Ponceau S-stained membrane. P-Pil1, phosphorylated Pil1. (B) Immunoblots for all phosphatase mutants were qualitatively scored based on their Pil1 phosphorylation phenotype in both glucose (green) and raffinose (purple) conditions compared with wild-type controls. The highest scoring mutants are indicated in red text.

**Fig. 5.**
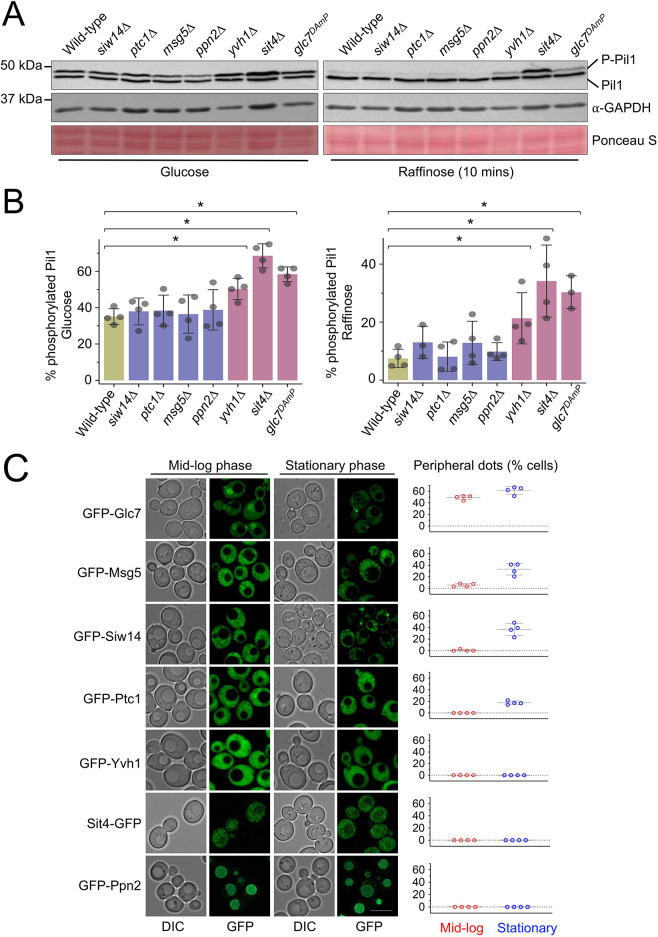
**Secondary activity and localisation screens of phosphatase candidates.** (A) Whole-cell lysates of wild-type and phosphatase mutant candidates from glucose-replete medium (left) or following 10 min exchange with raffinose medium were generated and analysed by immunoblotting using anti-Pil1 and anti-GAPDH antibodies. Ponceau S stain shown as an additional loading control. P-Pil1, phosphorylated Pil1. (B) Quantification of the percentage Pil1 phosphorylated in indicated mutants was calculated (*n*=four experiments, each quantifying >23 cells). (C) Yeast expressing GFP-tagged phosphatases were cultured to mid-log and stationary phase prior to confocal microscopy (Airyscan). The number of peripheral dots per cell (*n*>30) from separate experiments (*n*=4) were quantified in each condition (right). Scale bar: 5 µm.

As transporters are sequestered in eisosomes when Pil1 is dephosphorylated in response to glucose starvation, we hypothesise that Pil1 dephosphorylation plays a role in retaining nutrient transporters. To test this idea, we first set out to identify any responsible phosphatase enzymes and then test whether they have an impact on eisosomes, nutrient transporters or starvation recovery. In *S. cerevisiae*, 43 phosphatases have been identified (Offley and Schmidt, 2019), of which 39 are non-essential and four are essential. To identify the phosphatase(s) responsible for Pil1 dephosphorylation, we screened mutants of all phosphatase enzymes for their activity in glucose and raffinose conditions (Fig. 4A). Pil1 phosphorylation status was assessed in null mutants (Δ) for non-essential phosphatases or in cells with reduced expression of essential phosphatases, achieved by use of a DAmP cassette (Breslow et al., 2008). Mutants were scored based on defects in Pil1 dephosphorylation (Fig. 4B). This screen revealed seven top-scoring phosphatase mutants selected for further quantitative analysis (Fig. 5A,B). Next, we assessed the localisation of many GFP-tagged phosphatases at mid-log phase and stationary phase (Fig. 5C; Fig. S4). We included stationary phase as a nutritional stress associated with eisosome transporter retention (Gournas et al., 2018b). This confirmed a range of localisations, many to the nucleus and cytoplasm, but also Ppn2 at the vacuole and Ptc5 at the mitochondria (Breker et al., 2013). Interestingly, we also observed several GFP-tagged phosphatases that had changes in localisation following growth to stationary phase, including Sdp1, Ptc5 and Nem1. Although GFP–Msg5 and GFP–Siw14 fusions localised to the periphery upon growth to stationary phase, deletion of these mutants had no impact on Pil1 phosphorylation, so we assume this peripheral localisation is not related to eisosomal regulation. However, GFP–Glc7 showed significant peripheral punctate localisation in both growth conditions, in addition to localisation to the mid-body, the cytoplasm and the nucleus (Bloecher and Tatchell, 2000; Breker et al., 2013). The only phosphatase that showed significant localisation to the cell periphery and that caused defects in Pil1 phosphorylation upon mutation was Glc7.

### Glc7 controls dephosphorylation of Pil1

**Fig. 6.**
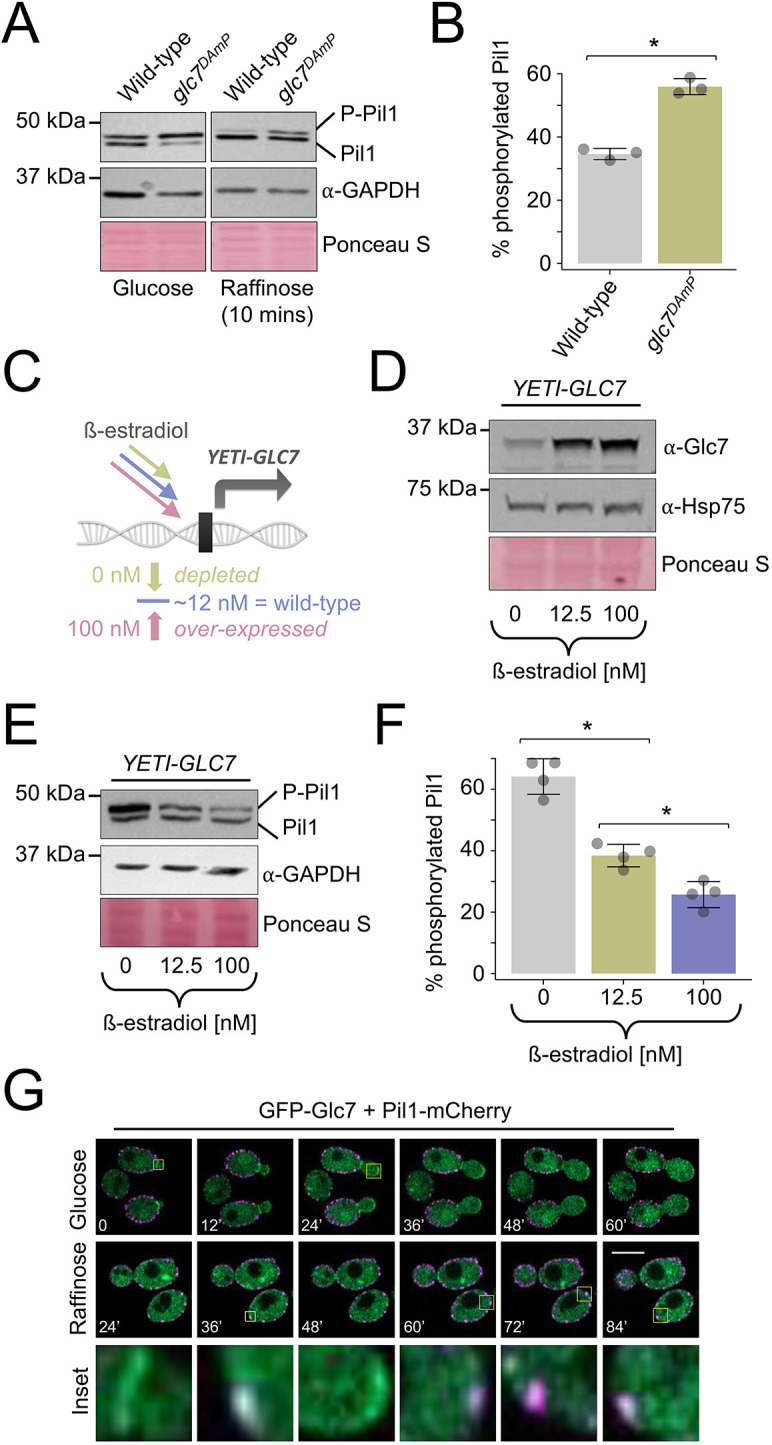
**Glc7 mediates dephosphorylation of Pil1.** (A) Wild-type and *glc7^DAmP^* cells were cultured in glucose medium and following 10 min of raffinose treatment prior to the generation of whole-cell lysates and immunoblotting with anti-Pil1 and anti-GAPDH antibodies. Ponceau S stain shown as an additional loading control. P-Pil1, phosphorylated Pil1. (B) The percentage of phosphorylated Pil1 from A was quantified (*n*=3). (C) Schematic outlining the principle of the YETI expression system for titratable expression of *GLC7* to mimic severely depleted (green) and over-expressed (pink) levels. (D,E) *YETI-GLC7* cells were grown overnight in 12.5 nM β-estradiol before washing three times in YPD medium and dilution in fresh medium containing 0, 12.5 and 100 nM β-estradiol. Cells were then grown for 6 h prior to the generation of whole-cell lysates and immunoblots using (D) anti-Glc7 and (E) anti-Pil1 antibodies. Loading controls using anti-GAPDH antibodies and Ponceau S-stained membrane are included for each. (F) Pil1 phosphorylation was quantified (*n*=4) from β-estradiol titrations shown in E. (G) Time-lapse microscopy of GFP–Glc7- and Pil1–mCherry-expressing cells was performed in glucose medium and following 25 min raffinose medium, with indicated time slices labelled (minutes). Images representative of three experiments. Scale bar: 5 µm. Quantitative results are mean±s.d. *P<0.05 (unpaired *t*-test).

Glc7 is an essential phosphatase (Clotet et al., 1991; Feng et al., 1991) that has been previously shown to function in glucose-related pathways, where it acts with its regulatory subunit Reg1 (Tu and Carlson, 1995), and in bud neck formation (Larson et al., 2008), among other roles in the cell. We confirmed that *glc7^DAmP^* results in reduced dephosphorylation of Pil1 in both glucose-replete and glucose starvation conditions (Fig. 6A,B). As a complementary approach to test the role of Glc7 in Pil1 phosphorylation, we altered *GLC7* expression levels using a yeast estradiol with titratable induction (YETI) strain (Arita et al., 2021), which allows modulation of expression by varying β-estradiol concentrations (Fig. 6C). We first show that protein levels of Glc7 can be controlled by exogenous β-estradiol concentrations (Fig. 6D), and this is specific to the *YETI-GLC7* strain (Fig. S5A,B). We then demonstrate that altering Glc7 levels, using a gradient of 0 nM, 12.5 nM and 100 nM β-estradiol, correlates with Glc7 activity in dephosphorylating Pil1 in a concentration dependent manner (Fig. 6E,F; Fig. S5C). We then tested whether GFP–Glc7 localised to eisosomes marked with Pil1–mCherry. Although, neither steady-state nor time-lapse imaging in glucose and raffinose media revealed large amounts of Glc7 localisation to eisosomes, there were often small regions of colocalisation at some eisosomes (Fig. 6G; Fig. S5D). This combinatorial approach strongly suggests that Glc7 is responsible for dephosphorylation of Pil1, and this might be achieved via Glc7 directly at eisosomes.

### Phosphorylation of Pil1 is important for starvation recovery

**Fig. 7.**
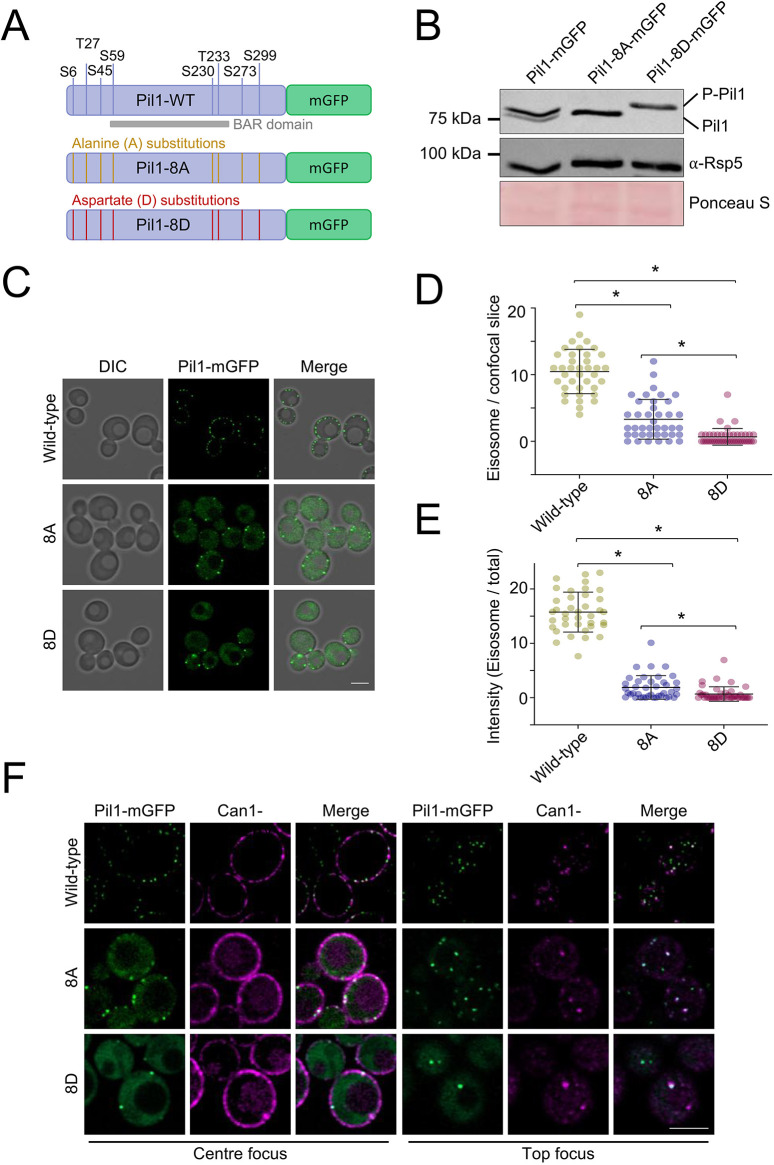
**Characterisation of Pil1 phospho-mutants.** (A) Illustration showing Pil1 fusion to mGFP, including its verified phospho-sites and BAR domain. Pil1 cassettes that have been mutated to give alanine (yellow) or aspartate (red) residues that were stably integrated at the *PIL1* locus are also shown. (B) Pil1 or indicated 8A and 8D mutants were expressed from the endogenous locus as mGFP fusions in strains grown to mid-log phase, harvested and lysate generated for immunoblotting with α-Pil1 and α-GAPDH antibodies (representative of *n*=3). Ponceau S stain included as an additional control. P-Pil1, phosphorylated Pil1. (C) Versions of mGFP-tagged Pil1 were expressed as the sole chromosomal copy and localisation was assessed by confocal microscopy (Airyscan 2). (D) Pil1-labelled eisosomes were identified by otsu segmentation and number per centre focussed confocal slice quantified (*n*≥37). (E) Integrated density for all GFP-tagged Pil1 versions localised to eisosomes identified from segmentation performed in D was calculated as a percentage of the total signal. (F) Wild-type and the phospho-mutants expressing Can1–mCherry were imaged using confocal microscopy (Airyscan 2). Micrographs show centre and top focus. Images representative of three experiments. Quantitative results are mean±s.d. *P<0.05 (unpaired *t*-test). Scale bars: 5 µm.

Having implicated Glc7 in Pil1 dephosphorylation, which occurs following glucose starvation, we next wanted to test whether this was connected to nutrient transporter residence in eisosomes and starvation recovery. Previous studies have assessed the phosphorylation profile of various Pil1 mutants with mutation of eight verified phospho-sites (Luo et al., 2008; Walther et al., 2007). We generated phospho-ablative (changed to alanine, 8A) and phospho-mimetic (changed to aspartate, 8D) versions of Pil1 at these phospho-sites (Fig. 7A). Western blotting confirmed that the 8A and 8D mutations resulted in Pil1 migrating not as a doublet, but as a single band, with faster migration of the phospho-ablative Pil1-8A–mGFP and slower migration of the phospho-mimetic Pil1-8D–mGFP fusion (Fig. 7B). Fluorescence microscopy of these mGFP-tagged Pil1 versions showed both 8A- and 8D-expressing strains exhibited an altered localisation phenotype compared to wild-type cells (Fig. 7C), as previously documented for phospho-mutants of Pil1 (Luo et al., 2008; Walther et al., 2007). We quantified these differences (Fig. S6A), revealing both mutants have fewer eisosomes compared to wild type (Fig. 7D) in addition to more cytoplasmic signal (Fig. 7E). This analysis showed a more pronounced defect in eisosome number and levels for Pil1-8D–mGFP than Pil1-8A–mGFP. To understand how nutrient transporter localisation might be affected in strains expressing phospho-mutants, we expressed the arginine transporter Can1, a dual reporter for eisosome morphology and transporter localisation. As expected, the abnormal localisation of Pil1–mGFP phospho-mutants is mirrored by Can1–mCherry, with fewer eisosome foci that colocalise with Pil1-8A and Pil1-8D (Fig. 7F). Although Can1–mCherry still localises to the PM when Pil1-8A and Pil1-8D are expressed, the punctate eisosome pattern is reduced and there is a small amount mis-localised to the vacuole (discussed in next section).

**Fig. 8.**
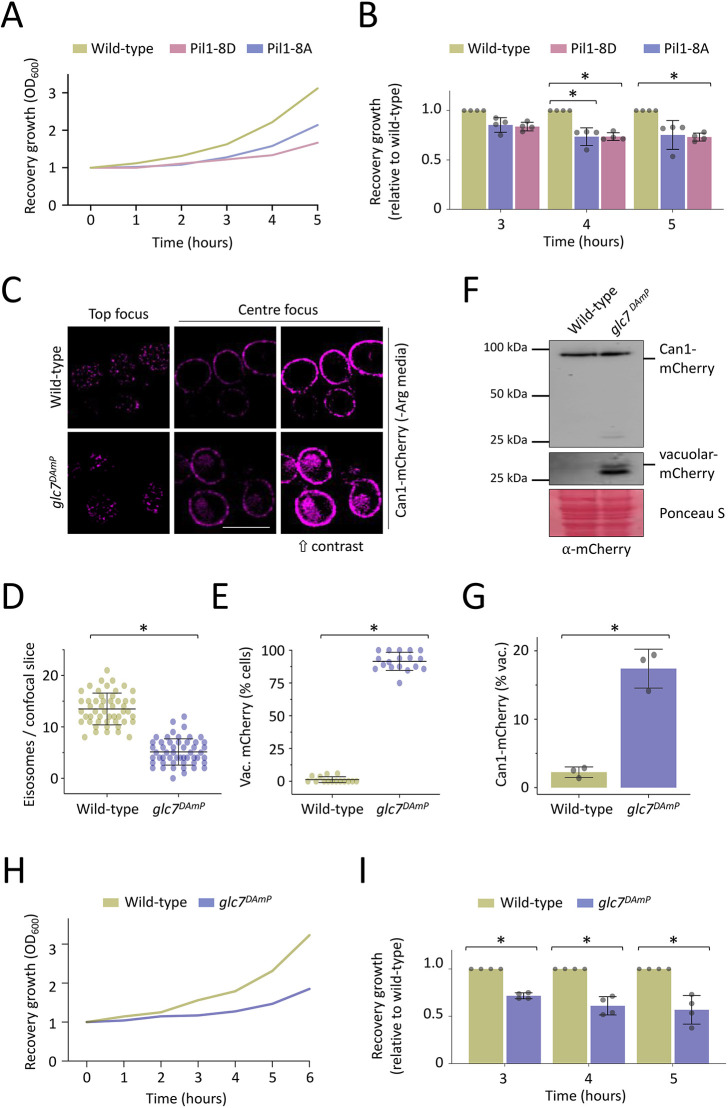
**Glc7 is required for efficient recovery from glucose starvation.** (A) Cells at mid-log phase were subjected to 2 h of glucose starvation (raffinose treatment), returned to glucose-replete conditions and growth measured over time. (B) The growth assay in A was repeated (*n*=4) and the growth relative to wild-type was quantified for each indicated time-point. (C) Wild-type and *glc7^DAmP^* cells endogenously expressing Can1–Cherry were grown in SC medium lacking arginine and imaged using confocal microscopy (Airyscan 2). Micrographs show top and centre focus, with increased contrast of the latter to show intravacuolar signal. Scale bars: 5 µm. (D) Eisosomes from top-focussed confocal slices were quantified (*n*>51). (E) Cells exhibiting intravacuolar (vac.) mCherry signal were quantified from centre focussed micrographs (*n*≥260 cells with averages from ∼15 micrographs plotted as a percentage). (F) Wild-type and *glc7^DAmP^* cells expressing Can1–mCherry were grown to mid-log phase, harvested and lysates were generated before immunoblotting using anti-mCherry antibodies. Full length Can1–mCherry and vacuolar processed mCherry fragment are indicated, including enhanced exposure of the latter. Ponceau S stain included as a loading control. (G) Densitometry was used to quantify the amount of vacuolar-processed mCherry from experiments in F (*n*=3). (H) Cells at mid-log phase were subjected to 2 h of glucose starvation (raffinose treatment) and then returned to glucose replete conditions, and growth measured over time. (I) The growth assay in H was repeated (*n*=4) and growth relative to wild-type was quantified for the indicated time-points. Quantitative results are mean±s.d. *P<0.05 (unpaired *t*-test).

To test whether Pil1 phospho-mutants were functional in starvation recovery, we used an assay that monitors recovery growth (Laidlaw et al., 2021). Although a subtle growth defect is observed for both Pil1-8A and Pil1-8D in rich medium, growth was indistinguishable to wild-type cells in SC minimal medium (Fig. S6B). All cells were cultured to log phase in SC medium followed by a 2-h glucose starvation. Upon a return to replete SC medium, we find that both Pil1-8A and Pil1-8D mutations are defective in recovery growth compared with wild-type cells (Fig. 8A,B). To test whether these results can be explained in the context of deregulation of nutrient transporters via Glc7 activity, we expressed Can1-mCherry in wild-type and *glc7^DAmP^* cells. These imaging results showed eisosome biogenesis still occurs in cells with less Glc7, but there was a significant reduction in eisosome number (Fig. 8C,D). Increased contrast of micrographs and quantification revealed that most *glc7^DAmP^* cells expressing Can1–mCherry, mislocalise a portion to the vacuole (Fig. 8C,E), much like phospho-mutant versions of Pil1. We also assessed Can1–mCherry mislocalisation biochemically, by immunoblotting cells and measuring stable mCherry following proteolytic processing in the vacuole. In agreement with the micrographs, essentially no Can1–mCherry is found in the vacuole in wild-type cells but a significant intravacuolar population is observed upon depletion of Glc7 (Fig. 8F,G). In support of our hypothesis that the described effects on eisosomes and nutrient transporter trafficking caused by depletion of Glc7 affect physiology following starvation, we used the recovery assay to show that despite no growth defects of *glc7^DAmP^* (Fig. S6C), the ability of *glc7* mutants to recover efficiently from glucose starvation is hampered (Fig. 8H,I).

Catalytic subunits of type 1 protein phosphatase (PP1) enzymes are known to co-function with a plethora of non-catalytic subunits, which can regulate specificity and localisation of enzymes (Virshup and Shenolikar, 2009). The best characterised regulatory subunit of Glc7 is Reg1, which is required for glucose repression (Tu and Carlson, 1995). Reg1 also has a paralogue Reg2, that is not involved in glucose repression but functionally complements the growth defect of *reg1*Δ cells (Frederick and Tatchell, 1996). However, deletion of either *REG1* or *REG2* alone or in combination had no significant effects on Pil1 phosphorylation (Fig. S7A,B). Genetic approaches have identified many factors thought to co-function with Glc7, with several predicted to be regulatory subunits (Logan et al., 2008). However, genetic mutation of these nine factors did not affect Pil1 phosphorylation (Fig. S7C,D). We then speculated that if an unknown factor existed, it might physically bind both Pil1 and Glc7. Bioinformatics revealed various candidates including some known to localise to the periphery (Fig. S8A). However, although Ygr237c localises quite prominently to eisosomes (Fig. S8B), deletion of these factors also failed to identify a Pil1 regulator in either glucose or raffinose conditions (Fig. S8C–F).

## DISCUSSION

Organisation of the yeast PM is complex, with various surface localisation patterns known for both integral membrane proteins and surface associated factors (Spira et al., 2012). Since the discovery of eisosome subdomains, progress has been made in understanding the formation and biological function of these structures, particularly in response to cellular stress (Babst, 2019; Megarioti et al., 2023 preprint; Moseley, 2018). The discovery that Pil1 and Lsp1, and their phosphorylation by the Pkh family kinases, are required for proper eisosome biogenesis (Walther et al., 2007, 2006) suggests that post-translational modification of core components could regulate the eisosome environment. Pkh1 and Pkh2 were originally identified as homologues of human and *Drosophila* 3-phosphoinositide-dependent protein kinase-1 (PDK1), which are essential for viability (Casamayor et al., 1999). As Casamayor et al. (1999) has demonstrated, the double *pkh1Δ pkh2Δ* yeast mutant is inviable, so a strain harbouring a temperature sensitive allele of *PKH1* (D398G) and deletion of *PKH2* (termed *pkh1^ts^ pkh2Δ*), was used to study kinase signalling pathways (Inagaki et al., 1999). This double *pkh1^ts^ pkh2Δ* mutant also revealed that there was an early association of Pkh kinases with endocytosis, as internalisation from the PM was impaired (Friant et al., 2001). Owing to the shared essential function of Pkh1 and Pkh2, the double mutant was the most logical strain to test effects on biogenesis of eisosomes (Walther et al., 2007). However, we clarify that Pkh2 is predominantly responsible for phosphorylating Pil1 with only minor roles for Pkh1 and the related kinase Pkh3, which also do not significantly localise to eisosomes (Fig. 1). One key difference to our work and a previous study that found Pkh1 at eisosomes (Walther et al., 2007) is that we did not use the *GAL1* promoter for over-expression, so it might be that the glucose starvation stress of galactose induction media alters Pkh1 localisation or the eisosome environment. Beyond this, Pkh2 expressed from its endogenous promoter is also found at eisosomes (Fröhlich et al., 2009). Exploring other potential kinases and phospho-sites revealed that the Hog1 and Cdc15 kinases might also have small roles regulating Pil1, but these effects were also relatively modest compared with Pkh2.

The finding that extracellular stress results in the accumulation of nutrient transporters in eisosome compartments, which deepen to facilitate this process, suggests a key role of eisosomes is related to nutrient uptake following stress (Appadurai et al., 2020; Gournas et al., 2018b). We have previously shown acute glucose starvation (2 h) results in concentration of the nutrient transporter Mup1 to eisosomes. As eisosomal mutants fail to properly retain nutrient transporters and fail to recover efficiently from starvation (Laidlaw et al., 2021), we propose acute glucose starvation modulates eisosomes to better harbour transporters for recovery. In this study, we show the core eisosomal protein Pil1 is rapidly dephosphorylated during this acute glucose starvation period, and systematic screening of enzyme activity and localisation identified the PP1 phosphatase Glc7 as a Pil1 modifier (Figs 3–5). This led to the model that the phosphorylation status of Pil1 is important for reorganisation of existing eisosomes during cargo retention, in addition to its established role in eisosome biogenesis. As membrane-bending effects of other BAR domain proteins are known to be affected by disordered regions (Busch et al., 2015; Zeno et al., 2018), it is conceivable that the charge and/or steric effects of Pil1 phosphorylation modulates its lipid binding/sculpting capacity, leading to nutrient transporter retention following starvation.

We also note that our screen identified significant effects on Pil1 dephosphorylation in both *sit4*Δ and *yvh1*Δ null strains. Sit4 is a PP2A type phosphatase first identified as regulating the cell cycle (Ronne et al., 1991). Since then it has been found to have many functional associations with nutrient signalling (Conrad et al., 2014). Sit4 has also been implicated in trafficking pathways used by nutrient transporters at the endoplasmic reticulum (Bhandari et al., 2013) and from endosomes to the surface (Amoiradaki et al., 2021) and vacuole (Gurunathan et al., 2002). Yvh1 is a phospho-tyrosine-specific enzyme (Guan et al., 1992) that is mainly associated with ribosome maturation (Kemmler et al., 2009) but has also been implicated in autophagy downstream of TORC1 following nutrient depletion (Yeasmin et al., 2015). Although beyond the scope of this work, it will be interesting to test whether these enzymes contribute to Pil1 dephosphorylation in response to nutrient starvation, either directly or indirectly. However, our screens identified the essential type 1 serine/threonine protein phosphatase Glc7 (Cannon et al., 1994; Peng et al., 1990) as the most likely candidate to regulate eisosomes via Pil1. Glc7 has several roles in the cell, including in growth, mitosis, transcription, stabilisation of emerging buds, glycogen metabolism and ion homeostasis (Feng et al., 1991; Hisamoto et al., 1995; Kozubowski et al., 2003; Peggie et al., 2002; Sanz et al., 2004; Williams-Hart et al., 2002), which we corroborate with expected localisations at the bud-neck, nucleus and cytoplasm (Fig. 5C). Our imaging experiments showed some occasions where Glc7 localises with Pil1 at eisosomes (Fig. 6G), so it might be that, at steady state, only a small percentage of Glc7 is required for eisosome maintenance, or this association could be transient. We confirmed Glc7 regulates Pil1 by depleting Glc7 using a ‘decreased abundance by mRNA perturbation’ (*DAmP*) method (Breslow et al., 2008) in addition to a yeast estradiol with titratable induction (YETI) depletion strategy (Arita et al., 2021), both of which showed elevated levels of Pil1 phosphorylation (Fig. 6). This latter approach allowed fine tuning of Glc7 protein levels, which coincided with predicted Glc7 activity levels. We have recently also used the YETI system to produce null, wild-type and mutant levels of the endosomal protein Ist1 (Laidlaw et al., 2022a). As YETI depletion and over-expression effects are induced within 6 h, this is an exciting alternative to genetic perturbations like CRISPR- or recombination-induced nulls, which take many generations to isolate a clonal population, allowing ample time for compensation to occur (El-Brolosy and Stainier, 2017).

As our phospho-ablative and phospho-mimetic versions of Pil1 were both defective, we assume being locked in either biochemical state does not promote eisosomal retention of transporters, but rather the fine-tuning of Pil1 phosphorylation is required to better harbour transporters acutely in response to nutritional stress. This mechanism could be important for understanding the metabolic response of yeast to varying nutrient conditions, including pathogenic fungi (Rutherford et al., 2019). Glc7 having a role in eisosomal modulation during glucose starvation is conceptually consistent with many studies demonstrating that Glc7 integrates with transcriptional repression in response to glucose availability, via Snf1 and downstream factors (Sanz et al., 2000; Tu and Carlson, 1994). However, as the changes we observe in both Pil1 dephosphorylation and transporter retention are very rapid, we assume these effects are not mediated at the transcriptional level. Other eisosome based regulation is known to span much longer periods of starvation (Gournas et al., 2018b; Megarioti et al., 2023 preprint) and likely to have transcriptionally based control. In further support of this being a novel and distinct role for Glc7, the regulatory subunit Reg1, which is required for many transcriptional related Glc7 activities (Alms et al., 1999; Cui et al., 2004; Dombek et al., 1999), or its paralogue Reg2 (Frederick and Tatchell, 1996), showed no increase in phosphorylated Pil1 species upon deletion (Fig. S8). Our additional efforts, both based on targets identified from the literature and through our own bioinformatic approaches, did not identify any other regulatory subunits of Glc7 involved with modifying Pil1. This might be explained by the fact that Glc7 has hundreds of potential regulators that have yet to be characterised (Logan et al., 2008; Ramaswamy et al., 1998), and the hypothetical possibility that some are functionally redundant.

Nonetheless, the fact that Glc7 is robustly associated with glucose metabolism via distinct mechanisms suggests there might be more complexity to the cellular response following starvation. It also remains to be understood how Glc7 senses glucose starvation prior to modifying eisosomes. One intriguing hypothesis is Glc7 activity is mediated via Pmp3, a cell periphery protein that is involved in maintenance of PM potential (Navarre and Goffeau, 2000). *pmp3*Δ mutants have defects in Pil1 phosphorylation and altered nutrient transporter stability, leading to the idea that Pmp3 is a phosphoinositide-regulated stress sensor (De Block et al., 2015). If so, this function might integrate with Glc7 modification of Pil1 following starvation, an idea supported by a genetic interaction between Glc7 and Pmp3 (Costanzo et al., 2010). Such mechanisms for eisosome regulation might also functionally connect with lipid homeostasis mediated by TORC2 (Riggi et al., 2018). This described role of stimuli-induced post-translational modification of lipid binding proteins, which triggers remodelling of membranes, might also apply to other compartments and other eukaryotic systems.

## MATERIALS AND METHODS

### Reagents

Table S2 documents yeast strains used in this study.

### Cell culture

Yeast cells were routinely grown in YPD (1% yeast extract, 2% peptone and 2% dextrose) or synthetic complete (SC) minimal medium (2% glucose, 0.675% yeast nitrogen base without amino acids, plus appropriate amino acid dropouts for plasmid selection) (Formedium, Norfolk, UK). 2% glucose was routinely used; where stated 4% glucose was used. Cells were subjected to glucose starvation using 2% raffinose rather than glucose as described previously (Laidlaw et al., 2021). Plasmid pCM1054 is a 2 µ over-expression plasmid for Pkh2 (Jones et al., 2008) used in Fig. 1E.

### Mating of yeast strains

Single colony haploid BY4741 mat **a** yeast strains encoding *URA3-*GFP-tagged genes (Weill et al., 2018) and BY4742 Mat **α** modified at indicated loci with mCherry-his5^+^ cassettes were isolated on YPD agar. Strain isolates were then mixed and mated on YPD agar, then single diploid colonies were isolated on SC medium lacking uracil and histidine, prior to confirmation of co-expression by fluorescence microscopy.

### Immunoblotting

Strains were grown to mid-log phase and equivalent volumes were harvested or starved for glucose with raffinose treatment prior to harvesting. Cells were treated to 0.2 N NaOH for 5 min prior to resuspension in lysis buffer (8 M urea, 10% glycerol, 50 mM Tris-HCl pH 6.8, 5% SDS, 0.1% Bromophenol Blue and 10% 2-mercaptoethanol). SDS-PAGE was used to resolve proteins which were then transferred to a nitrocellulose membrane using the iBlot dry transfer system (Invitrogen). Ponceau S strain was used to confirm successful transfer and equal loading. Membranes were probed with antibodies stated, details as listed in Table S3, and visualised using enhanced chemiluminescence (ECL) Super Signal Pico Plus (Thermo Fisher Scientific) and captured using a ChemiDoc Imager (Bio-Rad).

### Confocal microscopy

Yeast cells expressing fluorescently tagged proteins were grown to mid-log phase (unless stated) and then visualised in minimal medium at room temperature on Zeiss laser scanning confocal instruments (Zeiss LSM880 or Zeiss 980) using a 63×/1.4 objective lens. GFP was excited using a 488 nm laser and emission collected from 495 to 500 nm and mCherry was excited using the 561 nm laser and emission collected from 570–620 nm using an Airyscan (LSM880) or Airyscan 2 (LSM980) detector. Images were processed using Zeiss Zen software and modified (e.g. coloured) and quantified using ImageJ software (NIH).

### Recovery growth assays

Equivalent volumes of cells were harvested from mid-log cultures and washed three times with raffinose medium before being resuspended in raffinose medium and incubated in a shaking incubator at 30°C for 2 h. Equivalent volumes of the raffinose starved cells were harvested and washed three times with glucose medium before being resuspended in 100 µl of glucose medium. This was added to 3 ml of glucose medium and the optical density at 600 nm (OD_600_) was measured to obtain the value for time point 0. Subsequent OD_600_ measurements were taken every hour using a plate reader (Thermo Scientific) and normalised to values from wild-type cells.

### Spot growth assays

Equivalent volumes of indicated cells from mid-log phase cultures were harvested and a 10-fold serial dilution was created in water prior to spotting on solid agar medium plates. Plates were incubated at 30°C and images were captured at indicated time-points.

### Bioinformatic and statistical analyses

Prediction of kinase consensus motifs in Pil1 was undertaken by submitting the Pil1 amino acid sequence to the yeast database in Netphorest (Horn et al., 2014) and experimentally determined phospho-sites (Luo et al., 2008; Walther et al., 2007) were selected for analysis. Raw data for analyses is included in Table S1. The results were filtered with a minimum phosphorylation probability score of 0.1. Unlabelled immunoblots were assessed independently by two researchers (K.P. and C.M.). Any lanes perceived to have a defect in Pil1 phosphorylation received a score of +1, with a maximum score of 6 possible for any one mutant strain (i.e. deemed defective by both researchers in all three replicate experiments). The anonymised scores were then correlated to mutant strain labels and graphed. Unpaired two-tailed Student's *t*-tests were performed using GraphPad Prism v8.3.1. to compare the statistical significance between wild-type cells and mutants, or otherwise indicated pairwise comparisons, in described experimental conditions, with *P*-values documented in Table S4.

## Supplementary Material

10.1242/joces.260505_sup1 Supplementary information
