# Supplementary material for: The phosphatase Glc7 controls the eisosomal response to starvation via post-translational modification of Pil1
Source: J Cell Sci. 2023 Jul 24;136(14):jcs260505. doi: 10.1242/jcs.260505 (PMC10399984; doi:10.1242/jcs.260505)
Supplement: Supplementary information [file joces-136-260505-s1.pdf]

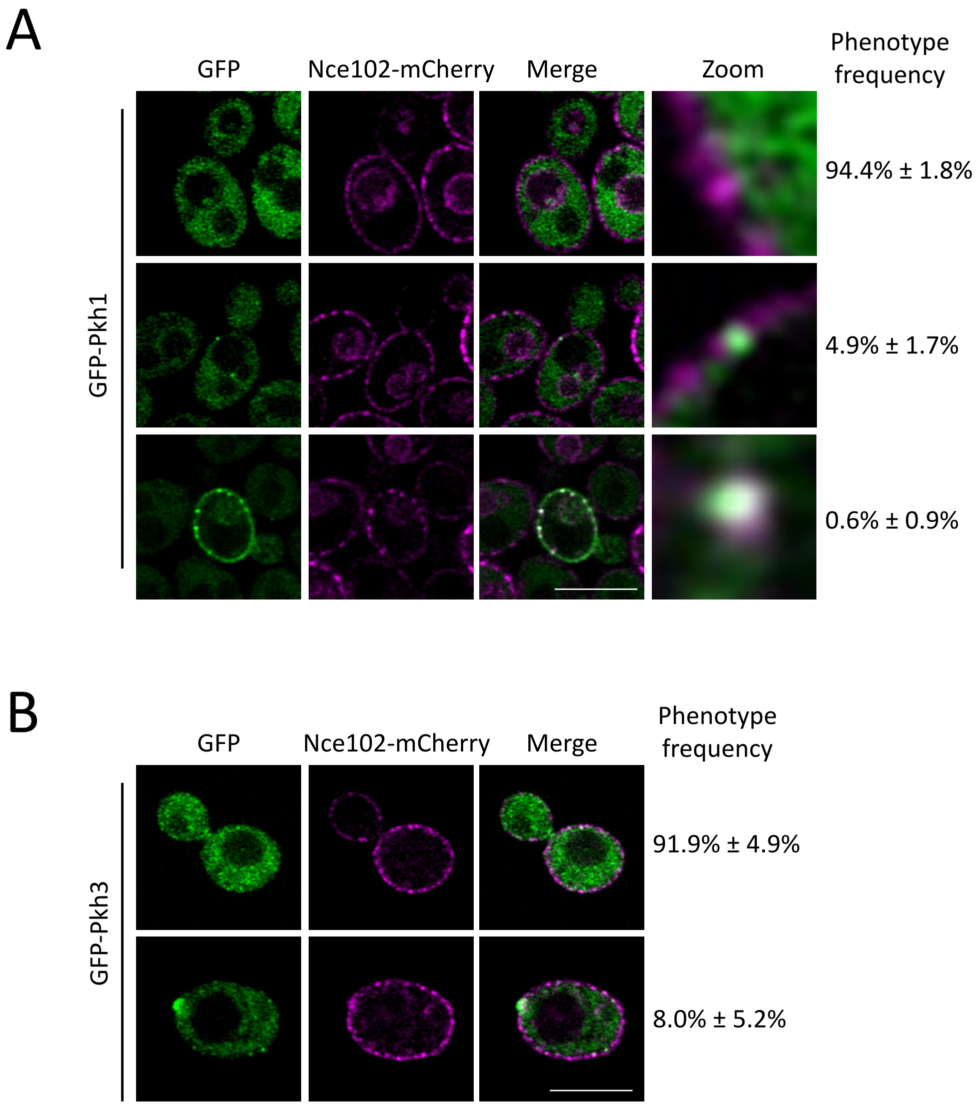

Fig. S1. Localisation phenotypes of Pkh kinases.

A - B) Wild-type cells co-expressing Nce102-mCherry with either GFP-Pkh1 (**A**) or GFP-Pkh3 (**B**) were imaged using confocal Airyscan 2 microscopy and the frequency of each phenotype observed was quantified. Scale bar = 5μm.

Journal of Cell Science • Supplementary information

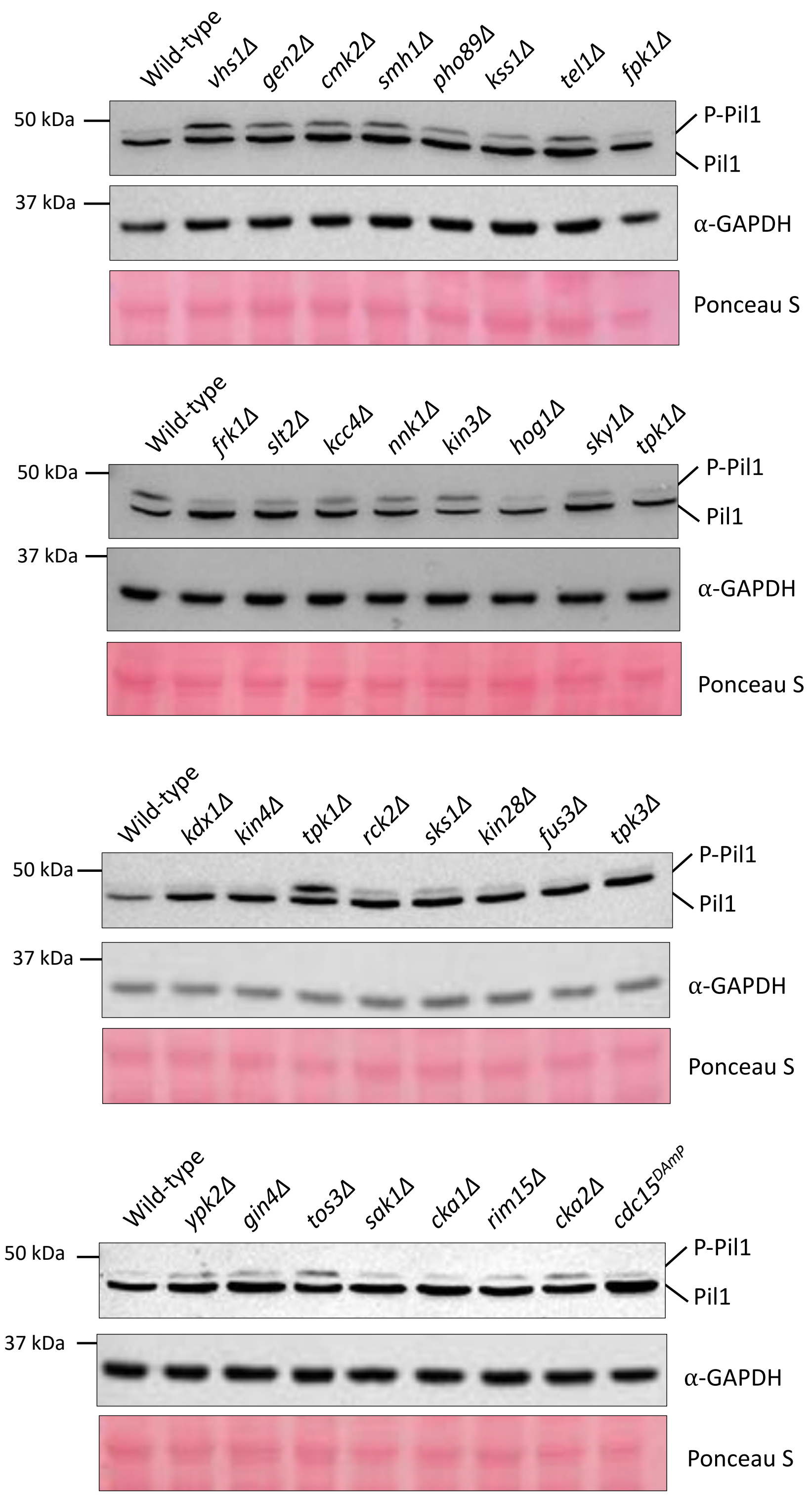

Fig. S2. Pil1 phosphorylation profiles of implicated kinase mutants.

Wild-type and mutant cells either lacking non-essential kinases (Δ) or with reduced expression of essential kinases (*DAmP*) were grown to mid-log phase and lysates were generated for immunoblot using α-Pil1and α-GAPDH antibodies. Ponceau S stained membranes are also provided as loading controls.

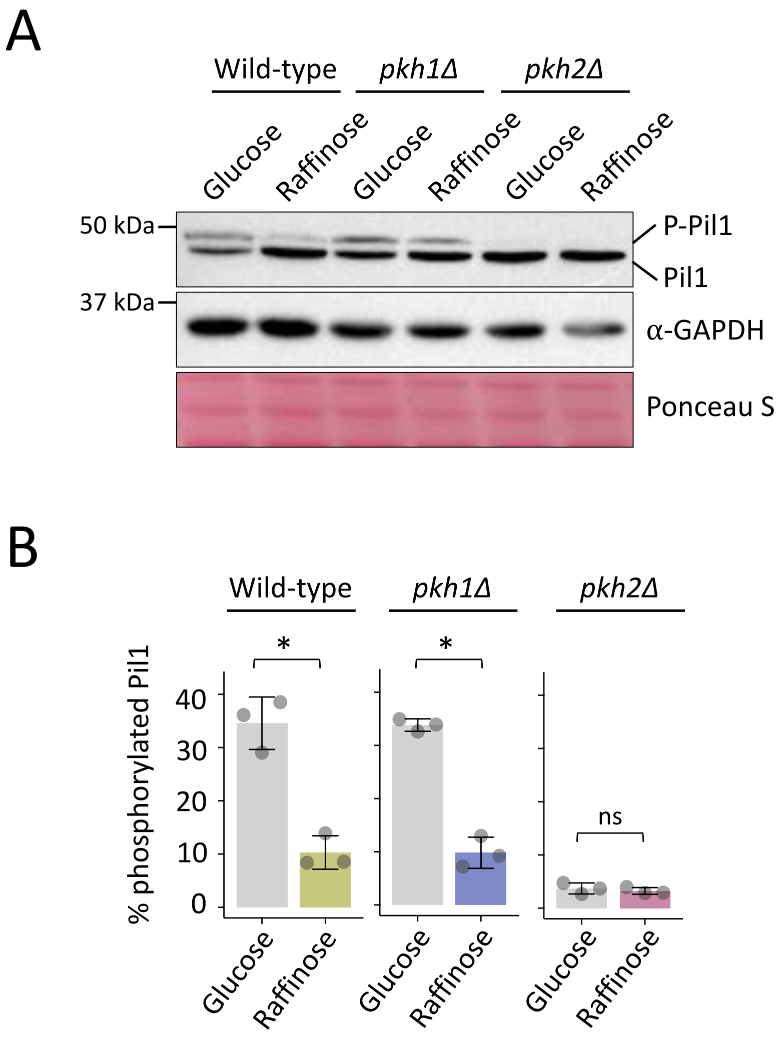

Fig. S3. Pil1 dephosphorylation in response to glucose starvation.

A) Whole cell lysates of wild-type, *pkh1*Δ and *pkh2*Δ cells in glucose media and after 10 minutes of raffinose treatment were analysed by immunoblotting using -Pil1 and -GAPDH antibodies; Ponceau S stained membrane is shown. **B)** The percentage of phosphorylated Pil1 from each strain was quantified and statistical significance was determined using a Student's t-test.

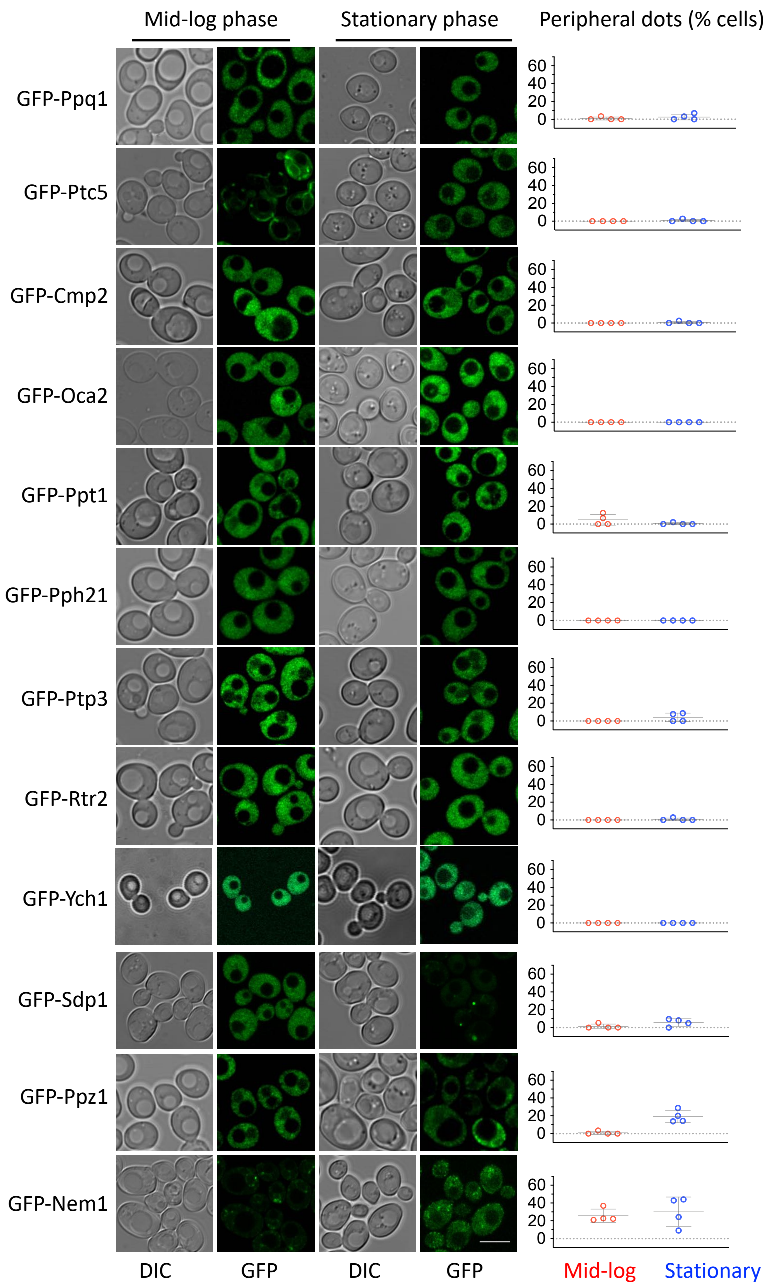

Fig. S4. Localisation of phosphatases at mid-log and stationary phase.

Indicated GFP tagged phosphatases were imaged using confocal Airyscan microscopy at mid-log and stationary phase. The number of peripheral dots per cell (n > 50) was quantified and the average for each experiment plotted (n = 4). Scale bar = 5μm.

Journal of Cell Science • Supplementary information

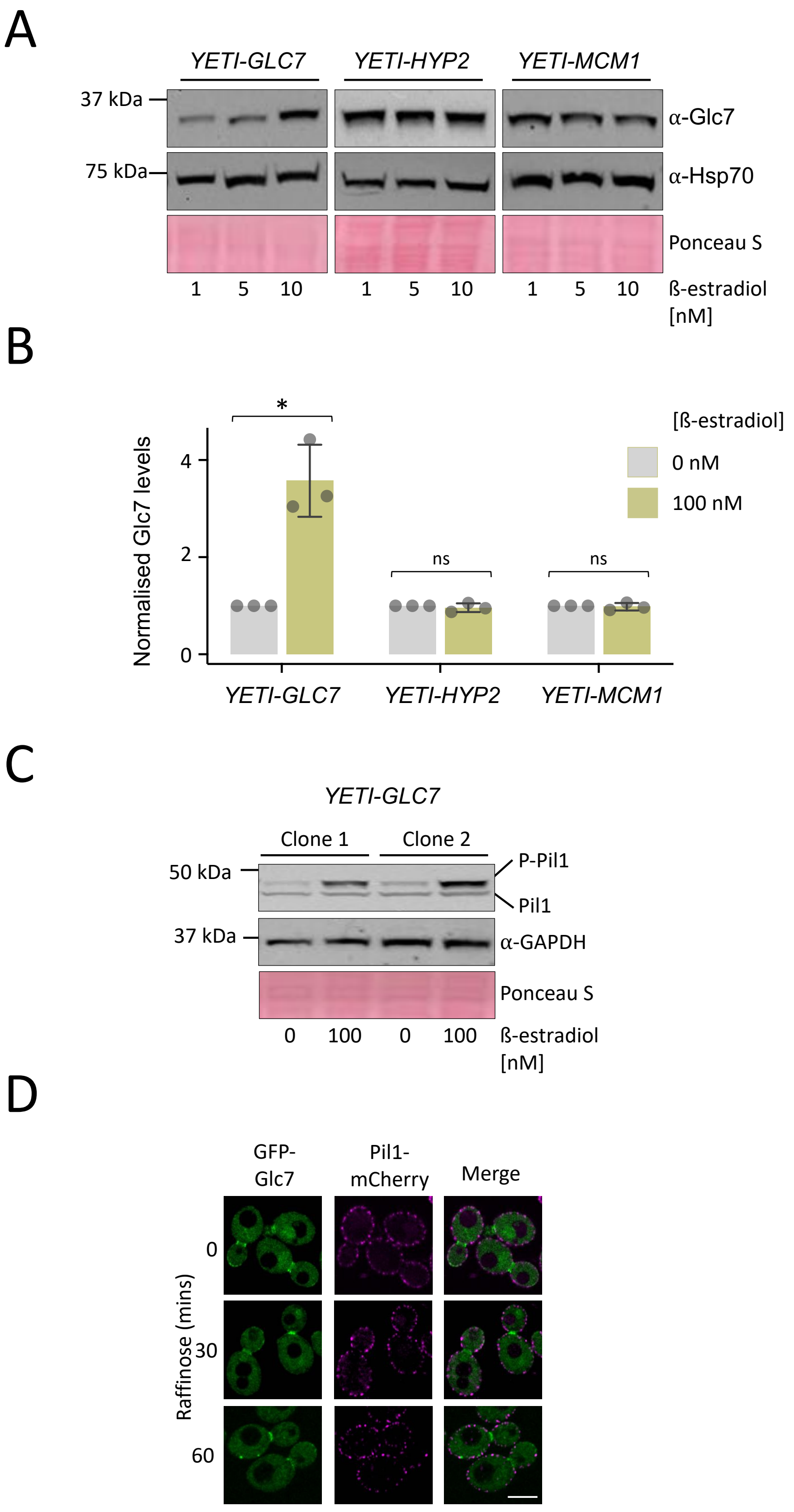

Fig. S5. Optimising Glc7 expression and localisation tools.

A) Indicated strains were grown overnight in YPD media followed 6 hour incubation with 1 nM, 5 nM and 10 nM β-estradiol prior to the generation of whole-cell lysates and immunoblots using α-Glc7 and α-Hsp70 antibodies. **B)** Levels of Glc7 from immunoblots normalised to loading were quantified. (n=3) with and without β-estradiol. **C)** *YETI-GLC7* cells were grown overnight in 0 and 100 nM β-estradiol prior to whole-cell lysate generation and immunoblots using α-Glc7 and α-GAPDH antibodies. **D)** GFP-Glc7 and Pil1-mCherry were grown to mid-log phase and imaged using confocal microscopy at 0, 30 and 60 minutes of raffinose treatment. Scale bar = 5μm.

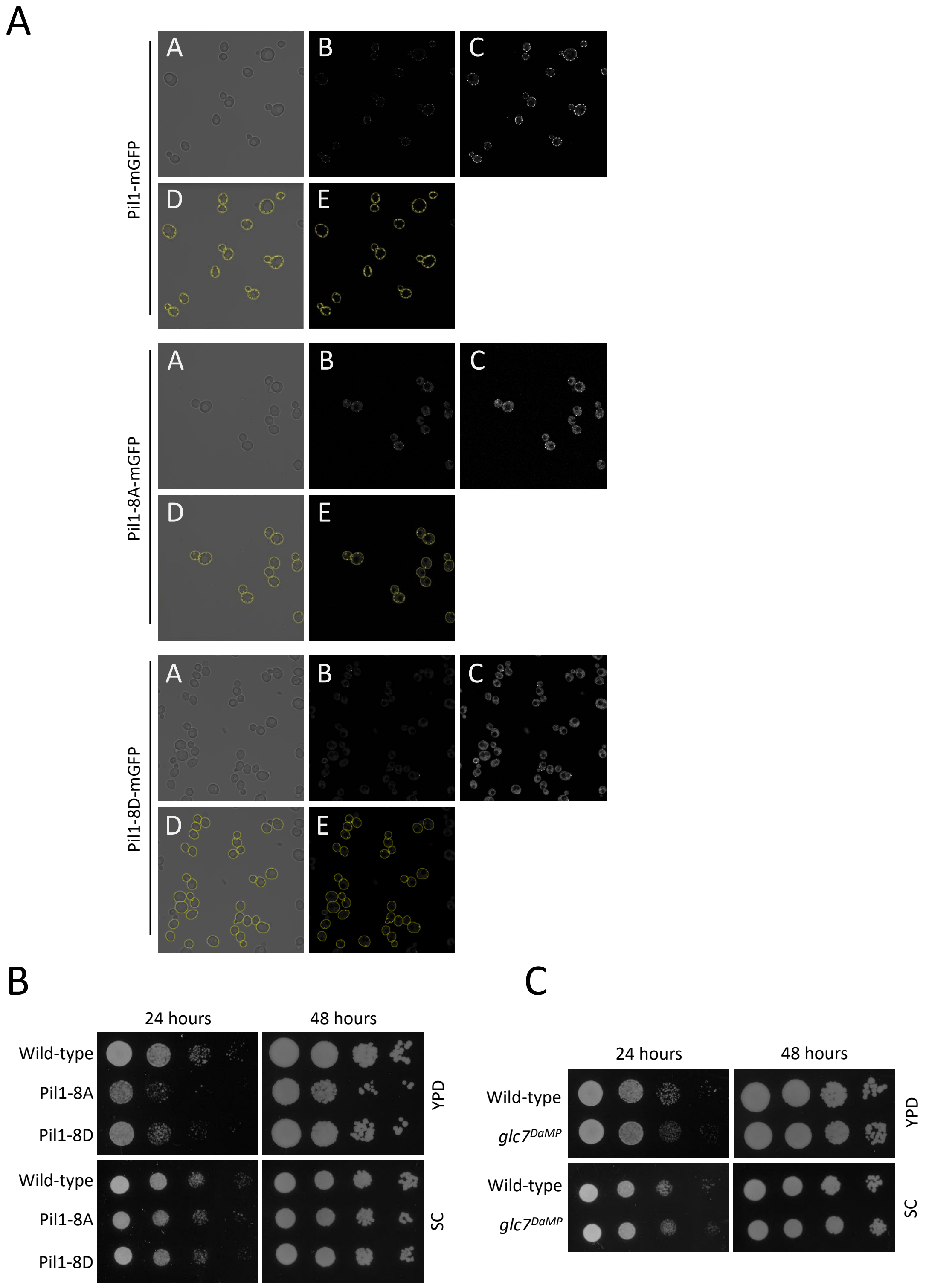

Fig. S6. Quantification method for Pil1 localisation phenotypes.

A) Versions of Pil1 tagged with mGFP were imaged using Airyscan 2 microscopy, brightfield (A) and fluorescence channels (B) are shown. A brighter version of GFP signal is included (C) to better demonstrate localisation distribution between eisosomes and the cytoplasm but not used for quantifications. Whole cell segmentation (A) was performed using Cell Magic Wand tool and combined with eisosome-only segmentation (D) using *ostu* thresholding using ImageJ. The number of eisosomes per cell were counted and the percentage of eisosome signal compared to total cellular signal was calculated from these regions of interest from each cell. Scale bar = 5µm. **B - C)** Wild-type cellular growth was compared to either Pil1-8A and Pil1-8D expressing cells **(B)** or *glc7^{DAmP}* mutants **(C)** by growing cells to mid-log phase in media (SC or YPD) before equivalent cell numbers were estimated by optical density measurements and harvested. 10-fold serial dilutions for each culture were generated, and yeast spotted out on both YPD plates and SC plates. Growth was recorded at 24 and 48 hours.

Journal of Cell Science • Supplementary information

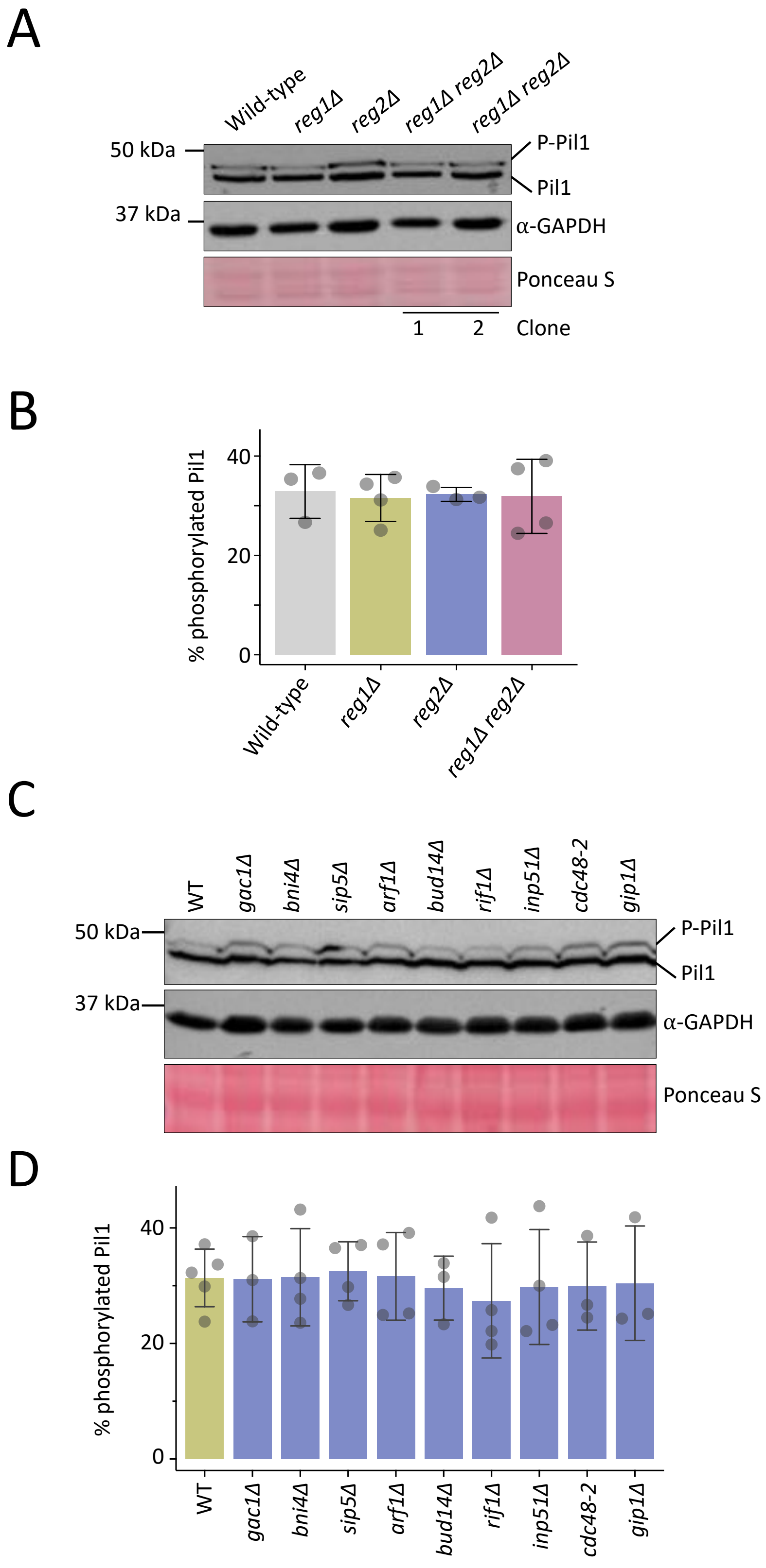

Fig. S7. Testing known Glc7 regulators for effect on Pil1 phophorylation.

A and C) Wild-type and mutant cells were grown to mid-log phase and lysates were generated for immunoblot using α-Pil1and α-GAPDH antibodies. Ponceau S stained membranes are also provided as loading controls. **B and D)** The percentage phosphorylated Pil1 from each yeast strain was quantified (n ≥ 3).

Journal of Cell Science • Supplementary information

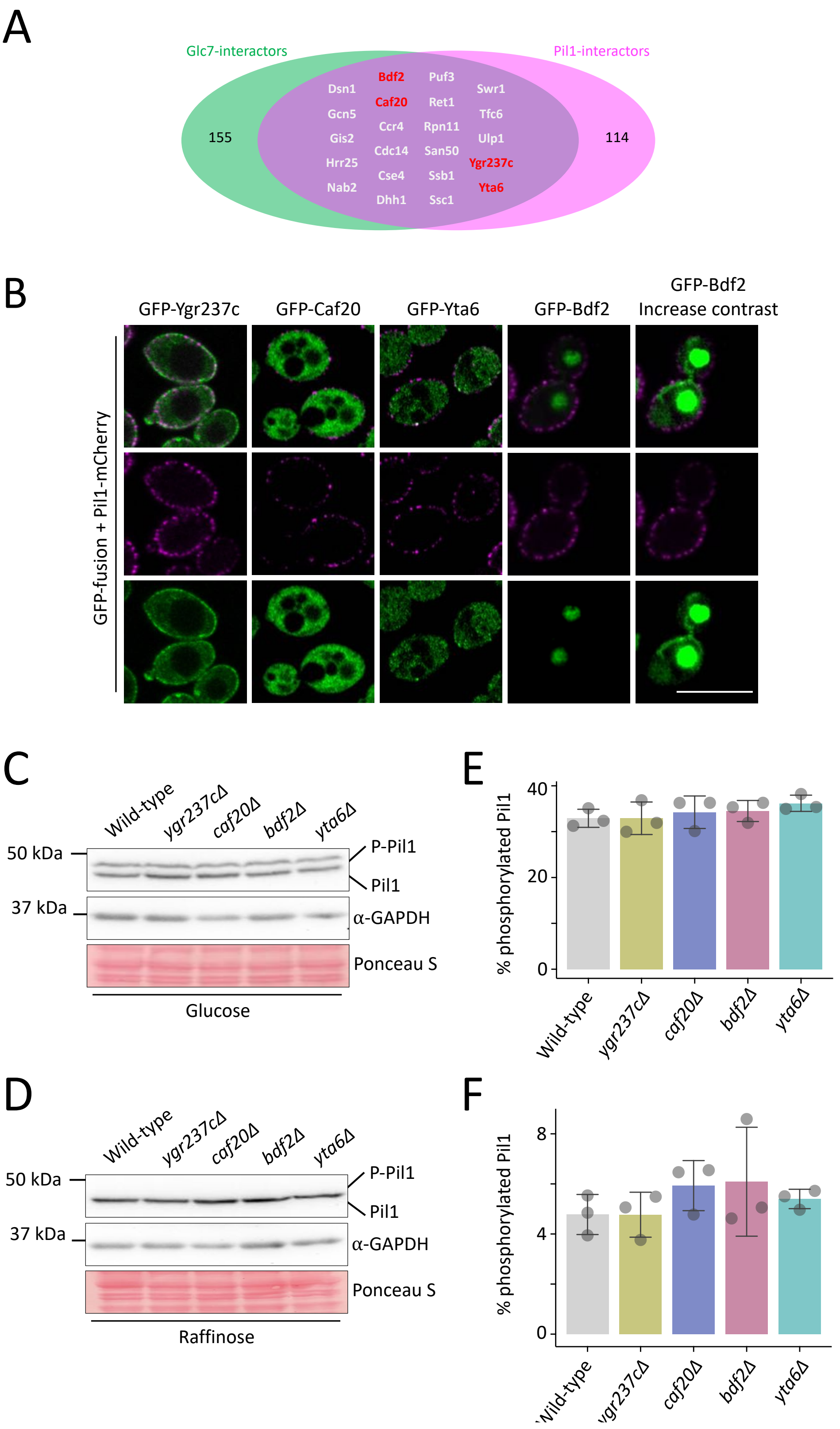

Fig. S8. Bioinformatic identification of potential Glc7 regulatory subunits

A) Venn diagram showing the overlap of Glc7 and Pil1 interactors. **B)** Indicated GFP-fusion strains were co-expressed with Pil1-mCherry and imaged at mid-log phase using confocal microscopy (Airyscan 2) to determine localisation. **C - D)** Wild-type cells and indicated mutants were grown to log phase in glucose replete (**C**) or subjected to 10 minutes raffinose treatment (**D**) conditions before equivalent volumes were harvested for immunoblotting with α-Pil1 and α-GAPDH antibodies. **E - F)**. The percentage phosphorylated Pil1 from each yeast strain (**C - D**) was quantified (n = 3). Statistical significance indicated (*). Scale bar = 5μm.

Journal of Cell Science • Supplementary information

Table S1. Netphorest hits

Click here to download Table S1

Table S2. Yeast Strains used in this study.

Click here to download Table S2

Table S3. Antibodies used in this study.

Click here to download Table S3

Table S4. Statistical analyses.

Click here to download Table S4

SUPPLEMENTAL REFERENCES

Arita, Y., G. Kim, Z. Li, H. Friesen, G. Turco, R.Y. Wang, D. Climie, M. Usaj, M. Hotz, E.H. Stoops, A. Baryshnikova, C. Boone, D. Botstein, B.J. Andrews, and R.S. McIsaac. 2021. A genome-scale yeast library with inducible expression of individual genes. *Mol Syst Biol*. 17:e10207.

Brachmann, C.B., A. Davies, G.J. Cost, E. Caputo, J. Li, P. Hieter, and J.D. Boeke. 1998. Designer deletion strains derived from Saccharomyces cerevisiae S288C: A useful set of strains and plasmids for PCR-mediated gene disruption and other applications. *In* Yeast. Vol. 14. 115-132.

Breslow, D.K., D.M. Cameron, S.R. Collins, M. Schuldiner, J. Stewart-Ornstein, H.W. Newman, S. Braun, H.D. Madhani, N.J. Krogan, and J.S. Weissman. 2008. A comprehensive strategy enabling high-resolution functional analysis of the yeast genome. *Nature Methods*. 5:711-718.

Laidlaw, K.M.E., D.D. Bisinski, S. Shashkova, K.M. Paine, M.A. Veillon, M.C. Leake, and C. MacDonald. 2021. A glucose-starvation response governs endocytic trafficking and eisosomal retention of surface cargoes in budding yeast. *Journal of Cell Science*. 134:jcs.257733-jcs.257733.

Peters, C., P.D. Andrews, M.J. Stark, S. Cesaro-Tadic, A. Glatz, A. Podtelejnikov, M. Mann, and A. Mayer. 1999. Control of the terminal step of intracellular membrane fusion by protein phosphatase 1. *Science*. 285:1084-1087.

Walther, T.C., P.S. Aguilar, F. Fröhlich, F. Chu, K. Moreira, A.L. Burlingame, and P. Walter. 2007. Pkh-kinases control eisosome assembly and organization. *EMBO Journal*. 26:4946-4955.

Weill, U., I. Yofe, E. Sass, B. Stynen, D. Davidi, J. Natarajan, R. Ben-Menachem, Z. Avihou, O. Goldman, N. Harpaz, S. Chuartzman, K. Kniazev, B. Knoblach, J. Laborenz, F. Boos, J. Kowarzyk, S. Ben-Dor, E. Zalckvar, J.M. Herrmann, R.A. Rachubinski, O. Pines, D. Rapaport, S.W. Michnick, E.D. Levy, and M. Schuldiner. 2018. Genome-wide SWAp-Tag yeast libraries for proteome exploration. *Nature methods*. 15:617-622.

Winzeler, E.A., D.D. Shoemaker, A. Astromoff, H. Liang, K. Anderson, B. Andre, R. Bangham, R. Benito, J.D. Boeke, H. Bussey, A.M. Chu, C. Connelly, K. Davis, F. Dietrich, S.W. Dow, M. El Bakkoury, F. Foury, S.H. Friend, E. Gentalen, G. Giaever, J.H. Hegemann, T. Jones, M. Laub, H. Liao, N. Liebundguth, D.J. Lockhart, A. Lucau-Danila, M. Lussier, N. M'Rabet, P. Menard, M. Mittmann, C. Pai, C. Rebischung, J.L. Revuelta, L. Riles, C.J. Roberts, P. Ross-MacDonald, B. Scherens, M. Snyder, S. Sookhai-Mahadeo, R.K. Storms, S. Véronneau, M. Voet, G. Volckaert, T.R. Ward, R. Wysocki, G.S. Yen, K. Yu, K. Zimmermann, P. Philippsen, M. Johnston, and R.W. Davis. 1999. Functional characterization of the S. cerevisiae genome by gene deletion and parallel analysis. *Science*. 285:901-906.
