# Supplementary material for: The phosphatase Glc7 controls the eisosomal response to starvation via post-translational modification of Pil1
Source: J Cell Sci. 2023 Jul 24;136(14):jcs260505. doi: 10.1242/jcs.260505 (PMC10399984; doi:10.1242/jcs.260505)
Supplement: Supplementary file 1 [file joces-136-260505_review_history.pdf]

Journal of Cell Science

The phosphatase Glc7 controls eisosomal response to starvation via posttranslational modification of Pil1

Katherine M. Paine, Kamilla M. E. Laidlaw, Gareth J.O. Evans and Chris MacDonald
DOI: 10.1242/jcs.260505

Editor: Michael Way

Review timeline

Original submission:	10 August 2022
Editorial decision:	6 September 2022
First revision received:	30 May 2023
Accepted:	22 June 2023

Original submission

First decision letter

MS ID#: JOCES/2022/260505

MS TITLE: The phosphatase Glc7 controls eisosomal response to starvation via posttranslational modification of Pil1

AUTHORS: Katherine M. Paine, Gareth J.O. Evans, Kamilla M. E. Laidlaw, and Chris MacDonald
ARTICLE TYPE: Research Article

We have now reached a decision on the above manuscript.

To see the reviewers' reports and a copy of this decision letter, please go to: https://submit-jcs.biologists.org and click on the 'Manuscripts with Decisions' queue in the Author Area. (Corresponding author only has access to reviews.)

As you will see, the reviewers have very different opinions, with one not recommending revision because of the lack of advancement. On balance, while they have raiseda number of substantial criticisms, I would be pleased to see a revised manuscript if you can address their concerns. I also think it is important to better highlight the advancement for the field in the MS and not just in the rebuttal letter to the reviewers. When you return therevised manuscript, we would then return it to the reviewers given their difference of opinion.

Please ensure that you clearly highlight all changes made in the revised manuscript. Please avoid using 'Tracked changes' in Word files as these are lost in PDF conversion.

I should be grateful if you would also provide a point-by-point response detailing how you have dealt with the points raised by the reviewers in the 'Response to Reviewers' box. Please attend to all of the reviewers' comments. If you do not agree with any of their criticisms or suggestions please explain clearly why this is so.

© 2023. Published by The Company of Biologists under the terms of the Creative Commons Attribution License (https://creativecommons.org/licenses/by/4.0/).

Reviewer 1

Advance summary and potential significance to field

Katherine Paine, Chris MacDonald and co-workers propose that the phosphatase Glc7 controls eisosomal response to starvation via posttranslation modification of Pil1 in the budding yeast S.cerevisiae.

In particular, they suggest that the phosphatase Glc7 is involved in the de-phosphorlyation of the major eisosome component Pil1 in response to glucose starvation. This Gcl7 dependent dephosphorylation of Pil1 is required for the rention of nutrient transporters (such as the methionine transporter Mup1) in eisosomes in response to glucose starvation. The retention of nutrient transporters in eisosomes ensures that the retained fraction of these transporters are protected from endocytosis. Therefore once glucose becomes available again, the retained transporters exit eisosomes, promote nutrient uptake and allow cells to quickly resume growth. Defects in this process hamper/delay cell growth and proliferation, because cells would loose too many nutrient transporters from the PM (by endocytosis and vacuolar degradation) and thus fail to import nutrients. While this model is certainly attractive the authors currently do to provide sufficient evidence to support their conclusion.

Comments for the author

Positive aspects of the manuscript:
1. The authors demonstrate clearly that Pil1 is dephosphorylated in response to glucose starvation.
2. The authors also identify several phosphatases (Yvh1, Sit4 and Glc7) that required to dephosphorylate Pil1.
3. Pil1-phospho mutants (both non-phosphorylatable (Pil1-8A) and phospho-mimetic (Pil1-8D) as well as Glc7 mutants, show defects in resuming cell growth after glucose starvation, supporting the idea that the phospho-regulation of eisosomes is important.

Major points:
1. The authors claim that cells with reduced Glc7 activity (and by extension with defects in Pil1 dephosphorylation) show defects in retaining Mup1 in eisosomes. The authors further propose that the failure to retain nutrient transporters in eisosomes leads to defects in resuming cell growth after glucose starvation. Unfortunately, the data presented in Fig8A is not sufficient to support the conclusion: (i) there are no obvious difference between WT and Glc7damp cells; (ii) there are no eisosme markers included, hence the claim that Mup1 is more - or less retained in eisosomes cannot be made (iii) moreover the differences may be subtle, and careful quantification will be required provided. The (possibly) reduced number of Mup1 'dots' may simply represent fewer eisosomes are formed in Glc7 mutants (as demonstrated for the Pil1-8A and the Pil1-8D mutant).
2. To substantiate these claims, similar experiments using co-localization of Mup1 with eisosomes should be conducted with Pil1-8A and Pil-18D mutants in response to glucose starvation and re-feeding (perhaps there is also a defect in releasing Mup1 from eisosomes once nutrient become available again?).
3. The author should test if the up-regulation of nutrient transporters (in response to nutrient availability: switch from raffinose to glucose) is hampered (or not) in the different mutants (particularly in the Glc7 mutant).
4. The growth defects of Pil1-8A and Pil1-8D mutant and of the Glc7 mutant are obvious, but they are difficult to interpret. It is not clear to me that they are causally linked to a failure in retaining nutrient transporters in eisosomes. Equally well they could be caused by defects in TORC2 signaling, mitotic progression or failure to up-regulate nutrient transporters.
5. Figure 3B: The authors claim that upon glucose starvation Mup1-GFP is concentrated in eisosomes, based on the co-localization of Mup1 with Pil1. I cannot see that. To me, the images show more co-localization of Mup1-GFP with Pil1 in cells growing in glucose. In the cells growing in raffinose, there appears to be less co-localization, but in these cells Mup1 is more frequently right next to Pil1.

© 2023. Published by The Company of Biologists under the terms of the Creative Commons Attribution License (https://creativecommons.org/licenses/by/4.0/).

Minor points:
Figure 1C: does GFP-PKH2 rescue the defects of a pkh1 mutant?
Figure 6: How do the protein levels change for Glc7 when using the YETI library?
To conclude that Glc7 function is indeed independent of Reg1, the paralog Reg2 should be tested as well (e.g.: in a reg1/2 double mutant…)
Fig 6: surely there must be more than 10 eisosomes on average / yeast (should the graph perhaps read eisosomes / cross section?)

Reviewer 2

Advance summary and potential significance to field

The present study identifies the kinase Pkh2 and the phosphatase Glc7 as major modifiers of the eisosomal protein Pil1. The nutrient status of the cell affects Pil1 phosphorylation, which in turn seems to affect the localization of nutrient transporters to eisosomes. The data suggest that the eisosomal storage of transporters observed during glucose starvation is dependent on the Glc7-mediated dephosphorylation of Pil1. Deletion of GLC7 results in a delayed recovery from starvation.

Comments for the author

The authors did a good job in quantifying the observations and in discussing the conclusions. However, the main issue is that there is not that much new insight into the regulation of eisosomes during starvation. Most key findings have been previously shown: the importance of eisosomes to deal with starvation, the storage of nutrient transporters in eisosomes, the importance of proper Pil1 phosphorylation for eisosome function and the involvement of the Pkh1/2/3 kinases in Pil1 phosphorylation. The manuscript presents new details in this context but conceptionally no new insight.

Specific comments:
- Loss of Sit4 has a strong effect on Pil1 phosphorylation. This is not further investigated because of the lack of Sit4 localization to the periphery. I am not convinced that the localization of the tested kinases is that meaningful since Pil1 is dynamically cycling on and off eisosomes and therefore some of the regulation might occur in the cytoplasm.
- The eisosome defect caused by the 8D or 8A mutant versions of Pil1 is severe and therefore it is not surprising that these mutants show similar starvation recovery defects as pil1 deletions.
- The manuscript states that transporters such as Mup1 are moving to eisosomes during glucose starvation. This is somewhat misleading since a large portion of nutrient transporters are endocytosed and degraded during starvation. Only a subset of the transporters remain at the surface and this subset localizes to eisosomes.

First revision

Author response to reviewers' comments

Reviewer Response Summary (authors comments in blue)

We thank both reviewers for their assessments, comments, and suggestions. We agree with Reviewer 1 that whilst the model is attractive our first submission lacked sufficient data to fully support it; chiefly, failing to show that mutants of Glc7 have a direct impact on nutrient transporters. To remedy this, we have taken various approaches to further strengthen the model, summarised below. One further issue is that our model is also based on previous studies, so we have clarified in more detail some critical results in the text. For example, Gournas and colleagues have shown different starvation responses, such as nitrogen depletion or extending growth past log phase, triggers sequestration of nutrient transporters in eisosomes (Figure provided for reviewer

© 2023. Published by The Company of Biologists under the terms of the Creative Commons Attribution License (https://creativecommons.org/licenses/by/4.0/).

has been removed - **Rebuttal Figure 1**). Also, our own work has previously shown that eisosomes efficiently harbour nutrient transporters during the first ~30 minutes of acute glucose starvation (Figure provided for reviewer has been removed - **Rebuttal Figure 2**).

Figure provided for reviewer has been removed - Rebuttal Figure 1: Cells expressing the Can1 nutrient transporter tagged with GFP were imaged confocal microscopy in rich media and also under starvation conditions induced by 1) growing cells in nitrogen free media for 2-hours and 2) extending the growth of cells past log phase. See Figure 5F from Gournas *et al*., 2018 PNAS (PMID: 29559531) https://doi.org/10.1073/pnas.1719462115

Figure provided for reviewer has been removed - Rebuttal Figure 2: Cells expressing the Mup1 nutrient transporter tagged were GFP was imaged by 4D Airyscan confocal microscopy during a microfluidic controlled media exchange from standard minimal media to a glucose starvation media. See Figure 5E and supplemental movie 5 from Laidlaw *et al*., 2021 J. Cell Sci. (PMID: 33443082) https://doi.org/10.1242/jcs.257733

Beyond these examples, work from the Babst lab further suggests nutrient transporter regulation occurs via eisosomes and plasma membrane tension, all controlled in response to stresses like starvation (Moharir *et al*,. 2018; Appadurai *et al*,. 2020). However, none of these studies provide a mechanistic explanation for changes in eisosomes that actively contribute to sequestration of a small reservoir pool of transporters at the plasma membrane. It is for this reason that we feel the assessment of reviewer 2 stating our study provides "no new insight" is not accurate. We hope our explanations in this response, and in the revised manuscript, do a better job at detailing 1) what was known previously, 2) what we have since clarified, 3) what we have discovered. We have undergone a substantial re-write and include much more detail for clarity and discussion. We have indicated below particularly relevant regions of text that have been modified. Our revised manuscript also now shows:

1) New data that shows the role of Glc7 dephosphorylation of Pil1 in regulating eisosomes ***and*** nutrient transporters (Figure provided for reviewer has been removed - **Figures 7F, 8C, 8D, 8E, 8F and 8G**).
2) Confirmation the YETI expression system can precisely modulate Glc7 protein levels (Figure provided for reviewer has been removed - **Figures 6D, S5A, S5B, S5C**).
3) Testing of the Reg1-Reg2 regulatory subunits of Glc7 to find neither, alone or in combination, are involved in Pil1 dephosphorylation (Figure provided for reviewer has been removed - **Figures S7A and S7B**). We test other known subunits (Figure provided for reviewer has been removed - **Figures S7C and S7D**).
4) Bioinformatic approaches that identified a range of additional candidates as potential Glc7 regulators, many of which we tested (Figure provided for reviewer has been removed - **Figures S8A - S8F**).

We have expanded the work significantly to address all the substantive issues (see below), and we hope this new work helps convince readers of the model, which we agree is an attractive explanation for this nutritional based physiological response and a new role for the PP1 phosphatase, Glc7.

Reviewer 1 - Specific Responses

Reviewer 1 Advance Summary and Potential Significance to Field:

Katherine Paine, Chris MacDonald and co-workers propose that the phosphatase Glc7 controls eisosomal response to starvation via posttranslation modification of Pil1 in the budding yeast S.cerevisiae.

In particular, they suggest that the phosphatase Glc7 is involved in the de-phosphorlyation of the major eisosome component Pil1 in response to glucose starvation. This Gcl7 dependent dephosphorylation of Pil1 is required for the rention of nutrient transporters (such as the methionine transporter Mup1) in eisosomes in response to glucose starvation. The retention of nutrient transporters in eisosomes ensures that the retained fraction of these transporters are protected from endocytosis. Therefore once glucose becomes available again, the retained

© 2023. Published by The Company of Biologists under the terms of the Creative Commons Attribution License (https://creativecommons.org/licenses/by/4.0/).

transporters exit eisosomes, promote nutrient uptake and allow cells to quickly resume growth. Defects in this process hamper/delay cell growth and proliferation, because cells would loose too many nutrient transporters from the PM (by endocytosis and vacuolar degradation) and thus fail to import nutrients. While this model is certainly attractive the authors currently do to provide sufficient evidence to support their conclusion.

Reviewer 1 Comments for the Author:

Positive aspects of the manuscript:

1. The authors demonstrate clearly that Pil1 is dephosphorylated in response to glucose starvation.
2. The authors also identify several phosphatases (Yvh1, Sit4 and Glc7) that required to dephosphorylate Pil1.
3.Pil1-phospho mutants (both non-phosphorylatable (Pil1-8A) and phospho-mimetic (Pil1-8D) as well as Glc7 mutants, show defects in resuming cell growth after glucose starvation, supporting the idea that the phospho-regulation of eisosomes is important.

We thank the reviewer for the summary of our discoveries. We also highlight the fact that clarifying the previously confused role of Pkh kinases in phosphorylation of Pil1 **(Figure provided for reviewer has been removed - Figure 1C - 1F, S1A - S1B, 2A - 2C, S2, S3A and S3B)** is an aspect of the work.

Major points:

1. The authors claim that cells with reduced Glc7 activity (and by extension with defects in Pil1 dephosphorylation) show defects in retaining Mup1 in eisosomes. The authors further propose that the failure to retain nutrient transporters in eisosomes leads to defects in resuming cell growth after glucose starvation. Unfortunately, the data presented in Fig8A is not sufficient to support the conclusion: (i) there are no obvious difference between WT and Glc7damp cells; (ii) there are no eisosme markers included, hence the claim that Mup1 is more - or less retained in eisosomes cannot be made (iii) moreover the differences may be subtle, and careful quantification will be required provided. The (possibly) reduced number of Mup1 ‘dots’ may simply represent fewer eisosomes are formed in Glc7 mutants (as demonstrated for the Pil1-8A and the Pil1-8D mutant).

(i) there are no obvious difference between WT and Glc7damp cells;

We agree the data of Mup1 localisation is difficult to unequivocally interpret, so we have removed this sub-panel. Although Mup1-GFP localises to eisosomes it is also found in multiple distinct subdomains of the PM (Busto *et al.*, **EMBO** 2018) and has the tendency to accumulate in endosomes following changes in nutrition (MacDonald *et al.*, **Dev. Cell** 2015). Instead, we created a whole series of new chromosomally tagged reagents that express the classical eisosome marker Can1, labelled with mCherry.

Using this optimised tool, we observed several phenotypes upon depletion of the essential Glc7 phosphatase. The number of eisosomes per cell (measured from top focus confocal images) marked with Can1 was reduced significantly (Figure provided for reviewer has been removed – **Figures 8D and 8E**). Furthermore, these experiments revealed a low-level signal of Can1 being sorted to the vacuole lumen in most cells upon depletion of Glc7 (Figure provided for reviewer has been removed – **Figures 8D, 8F**). We further validated this phenotype by biochemically assessing the amount of Can1-mCherry sorted to the vacuole (Figure provided for reviewer has been removed – **Figures 8G, 8H**), which can be quantified by the levels of proteolytically cleaved mCherry that traffics to the lumen of the vacuole.

(ii) there are no eisosme markers included, hence the claim that Mup1 is more - or less retained in eisosomes cannot be made

We thank the reviewer for these points. As described above, we took advantage of the arginine transporter Can1, which gives its name to the marker (Membrane comportment occupied by Can1, MCC) first used to define eisosomes (Malinska *et al.*, **Mol. Biol. Cell** 2003). This tool has the added benefit or localising more specifically to eisosomes than Mup1, whilst also being a nutrient

© 2023. Published by The Company of Biologists under the terms of the Creative Commons Attribution License (https://creativecommons.org/licenses/by/4.0/).

transporter. We made various attempts (using multiple strategies) to combine a *glc7* mutation in a dual tagged strain labelling both Pil1 and Can1, but this was unsuccessful. Irrespective, the analyses from all our successfully created yeast strains, and the extra observations about Can1 trafficking that could be followed up biochemically, do a much better job of validating the model.

(iii) moreover the differences may be subtle, and careful quantification will be required provided. The (possibly) reduced number of Mup1 'dots' may simply represent fewer eisosomes are formed in Glc7 mutants (as demonstrated for the Pil1-8A and the Pil1-8D mutant).

Good point, and indeed the differences although are obvious, they did benefit from more detailed quantitative analysis. By developing tools based on Can1-mCherry, we were able to observe eisosome morphology ***and*** the levels of Can1 downregulation are altered following *glc7* mutation, as described above in (1-i).

2. To substantiate these claims, similar experiments using co-localization of Mup1 with eisosomes should be conducted with Pil1-8A and Pil-18D mutants in response to glucose starvation and re-feeding (perhaps there is also a defect in releasing Mup1 from eisosomes once nutrient become available again?).

This was also a very useful comment, thank you. To address this point about eisosomes / transporters in the phospho-mutants, we created co-expressing strains of Pil1-mGFP (or mutants thereof) and Can1-mCherry, which were successful. This revealed that although there was not a complete loss of eisosomes or surface puncta in the phospho-mutants, eisosome morphology is severely abrogated. One would probably not expect complete loss of eisosomes, since even *pil1Δ* cells do not entirely lack them (Walther, **Nature** 2006, Vesela, **J. Cell Sci.** 2023).

In summary, these first two comments led us to reveal that that eisosomes regulate nutrient transporters directly via Glc7, which bolsters the model significantly.

3.The author should test if the up-regulation of nutrient transporters (in response to nutrient availability: switch from raffinose to glucose) is hampered (or not) in the different mutants (particularly in the Glc7 mutant).

Whilst such a transcriptional response might occur as an additional level of regulation, we assume the Pil1 dephosphorylation and nutrient sequestration we observe in the initial minutes following glucose starvation are not explained by such a mechanism. However, this does lead to an important point now included in our discussion that this mechanism should be considered in the context of an *acute* starvation response. We now also discuss this and cite a recent preprint (Megarioti *et al.*, **BioRxiv** 2023) that shows eisosomes regulate responses to much longer starvation as cells enter a quiescent programme, which would be much more likely to be regulated at the transcriptional level (**Page 4; line 25**).

4.The growth defects of Pil1-8A and Pil1-8D mutant and of the Glc7 mutant are obvious, but they are difficult to interpret. It is not clear to me that they are causally linked to a failure in retaining nutrient transporters in eisosomes. Equally well they could be caused by defects in TORC2 signaling, mitotic progression or failure to up-regulate nutrient transporters.

It is important to reinforce that these results do not represent "growth defects" in the traditional sense. Indeed, we ***do not*** observe any obvious differences in growth of wild-type cells with either the phospho-mutants of Pil1 or the *glc7* cells. Instead, the recovery assay measures growth ***following*** recovery (and return to replete conditions). We developed this assay for a previous study (Laidlaw *et al.*, **J. Cell Sci.** 2021) and have used it to show eisosome mutants (*eis1Δ*, *pkh2Δ* and *nce102Δ* mutants) have decreased "recovery growth". We then showed this effect could be ameliorated, but not fully rescued, by creating uracil prototroph strains (Laidlaw *et al.*, **J. Cell Sci.** 2021), linking the recovery effect directly to surface transporters like Fur4 being at the surface (Paine *et al.*, **Traffic** 2021). This is clarified in the Results section (**Page 4; line 43**) and by separating the growth assays (Figure provided for reviewer has been removed - **Supplemental Figure S6B - S6C**) from the recovery assays (Figure provided for reviewer has been removed - **Figures 8A - 8B, 8H - 8I**) and discussing separately.

© 2023. Published by The Company of Biologists under the terms of the Creative Commons Attribution License (https://creativecommons.org/licenses/by/4.0/).

5.Figure 3B: The authors claim that upon glucose starvation Mup1-GFP is concentrated in eisosomes, based on the co-localization of Mup1 with Pil1. I cannot see that. To me, the images show more co-localization of Mup1-GFP with Pil1 in cells growing in glucose. In the cells growing in raffinose, there appears to be less co-localization, but in these cells Mup1 is more frequently right next to Pil1.

The comparison of these two conditions is difficult, mainly because Mup1-GFP molecules are internalised and removed from diffuse surface regions (i.e. non-eisosomal) following starvation. We have therefore adjusted the GFP channels from these top focussed slices to be the same in glucose versus raffinose. In addition to explaining in the text that the important distinction is the concentration of molecules in an eisosome regions is much higher following starvation.
We believe the puncta in **Figure 3B** represent colocalizations between Pil1-mCherry and Mup1-GFP. This is in keeping with previous studies by us and others (see Figure provided for reviewer has been removed – **Rebuttal Figure 3**) that show a degree of distinction between transporters and Pil1, on account of their specific localisations and eisosomes being although small, at ~200-400nm they are above the diffraction limit for confocal microscopy.

Rebuttal Figure 3: Cells co-expressing transporter Mup1-GFP and eisosome marker Pil1-mCherry show localisation to same compartment does not result in perfect "colocalization." See Figure 6D from Laidlaw *et al.*, 2021 J. Cell Sci. (PMID: 33443082) https://doi.org/10.1242/jcs.257733

I suspect the images discussed in glucose are perceived to show "more colocalization" is due to these cells having similar eisosomal (albeit less GFP) signal ***in addition*** to the diffuse signal from non-eisosome PM regions. This artificially increases the perceived colocalization. For this reason, we now explain the data and interpretation very carefully (**Page 3; line 34**). We would also like to draw the reviewer's attention to the observation that transporters diffuse in and out of eisosomes - and somewhat surprisingly molecules in eisosomes prior to starvation are not necessarily the eisosomes that accumulate transporters (Figure provided for reviewer has been removed – **Rebuttal Figure 2 -** compare left cell and right cell before / after glucose starvation).

Minor points:

Figure 1C: does GFP-PKH2 rescue the defects of a pkh1 mutant?

The take home message from these figure subpanels is that Pkh2 is mainly responsible for Pil1 phosphorylation. This suggested experiment is unlikely to reveal interpretable data because 1) *pkh1Δ* mutants have a very small phenotype to rescue and 2) if phosphorylation was rescued by Pkh2 in a *pkh1* mutant this wouldn't necessarily be explained by functional complementation, since even in a wild-type cell Pil1 phosphorylation can be increased above basal by expression of Pkh2 (Figure provided for reviewer has been removed – **Figures 1E, 1F**).

Figure 6: How do the protein levels change for Glc7 when using the YETI library?

We acknowledge the deficiencies of the initial work that relied on indirect measures of Glc7 using the phosphorylation status of its substrate Pil1. To better address this we obtained an antibody and have now shown that the expression levels of Glc7 induced from estradiol titration correlates with protein levels (Figure provided for reviewer has been removed – **Figures 6C, 6D, S5A, S5B**) in addition to activity (Figure provided for reviewer has been removed – **Figure 6E, 6F, S5C**) of Glc7. Thank you for pointing this out as we consider this result a great validation of the YETI library at the protein level, which we were keen to document, as we believe this library to be a very important for yeast cell biology going forward. We now include a discussion of this and the issues when using CRISPR, etc to generate null mutants (**Page 6; line 15**)

To conclude that Glc7 function is indeed independent of Reg1, the paralog Reg2 should be tested as well (e.g.: in a reg1/2 double mutant…)

© 2023. Published by The Company of Biologists under the terms of the Creative Commons Attribution License (https://creativecommons.org/licenses/by/4.0/).

We thank the reviewer for this idea. We had not considered the paralogue Reg2 as a regulator of Glc7. We created single and double mutants to test this idea. However, there was no difference in Pil1 phosphorylation compared to wild-type cells in *reg1Δ*, *reg2Δ* or *reg1Δ reg2Δ* double mutants (Figure provided for reviewer has been removed - **Figures S7A - S7B**). This result might be expected, as although Reg2 binds Glc7 and *reg1Δ reg2Δ* cells have a synthetic genetic defect in growth (Frederick *et al*., **Mol. Cell Biol.** 1996), this study also showed that Reg2 overexpression cannot rescue glycogen accumulation or glucose repression of *reg1Δ* cells, which we now discuss (**Page 5; line 4**).

This question, and the experimental results described above, highlight a question that we tried to address: what other factors collaborate with Glc7 to mediate Pil1 phosphorylation? For these, we scoured the literature to identify candidates that might reduce Pil1 dephosphorylation upon their deletion/depletion. However, mutants representing all 9 candidates, did not affect Pil1 phosphorylation (Figure provided for reviewer has been removed - **Figures S7A and S7B**).

We then speculated that if an unknown factor existed, it would need to bind both Pil1 and Glc7. We performed bioinformatics to identify candidates for such a regulator (Figure provided for reviewer has been removed - **Figures S8A**) by virtue of known interactomes for Pil1 and Glc7. Although this effort yielded further candidates, even one uncharacterised protein (Ygr237c) that localises to eisosomes (**Figures S8B**), deletion of any of these factors also failed to identify a Pil1 regulator (Figure provided for reviewer has been removed - **Figures S8C - S8F)**. We now discuss why this might be (**Page 6; line 33**).

Fig 6: surely there must be more than 10 eisosomes on average / yeast (should the graph perhaps read eisosomes / cross section?)

Yes, this is a careless oversight that could lead to misunderstanding! We have altered the legend and axis label.

Reviewer 2 Advance Summary and Potential Significance to Field:
The present study identifies the kinase Pkh2 and the phosphatase Glc7 as major modifiers of the eisosomal protein Pil1. The nutrient status of the cell affects Pil1 phosphorylation, which in turn seems to affect the localization of nutrient transporters to eisosomes. The data suggest that the eisosomal storage of transporters observed during glucose starvation is dependent on the Glc7-mediated dephosphorylation of Pil1. Deletion of GLC7 results in a delayed recovery from starvation.

Reviewer 2 Comments for the Author:
The authors did a good job in quantifying the observations and in discussing the conclusions. However, the main issue is that there is not that much new insight into the regulation of eisosomes during starvation. Most key findings have been previously shown: the importance of eisosomes to deal with starvation, the storage of nutrient transporters in eisosomes, the importance of proper Pil1 phosphorylation for eisosome function and the involvement of the Pkh1/2/3 kinases in Pil1 phosphorylation. The manuscript presents new details in this context but conceptionally no new insight.

We respectfully disagree with this summary of our work and the implied novelty (or lack thereof) in the manuscript. For example, consider the statement “Most key findings have been previously shown” in the context of:

1. “The importance of eisosomes to deal with starvation”
Although this has been recently shown to occur for several transporters and starvation conditions, to my knowledge only we have shown transporter sequestration following acute glucose starvation. Our new data show this condition triggers changes in eisosome factor Pil1 at the posttranslational level, mediated by the phosphatases like Glc7 (that we identified from a systematic screen), all of which is conceptually novel. We then go on and show this is independent from canonical Glc7 functions and its established coregulators.

© 2023. Published by The Company of Biologists under the terms of the Creative Commons Attribution License (https://creativecommons.org/licenses/by/4.0/).

2. “the storage of nutrient transporters in eisosomes”
Yes, it has been shown that nutrient transporters are stored in eisosomes - and I believe we may be the only lab to demonstrate this process in real time (**Rebuttal Figure 2**). However, although this has been observed - almost zero information is known about how this is controlled at a mechanistic level. Our manuscript provides new understanding to how phosphorylation of BAR domain proteins organises subdomains of the plasma membrane.

3. “the importance of proper Pil1 phosphorylation for eisosome function”
Whilst Pil1 phosphorylation has been shown previously to be important **for eisosome biogenesis** it does not naturally follow that eisosome retention of nutrient transporters in response to starvation is also regulated by Pil1 phosphorylation status. We show for the first time Pil1 de/phosphorylation clearly also has a more nuanced regulatory role in eisosomes responding to starvation. This comment also does a disservice to our discovery that Pil1 dephosphorylation - not phosphorylation - is important for responding to glucose starvation. This is an entirely novel observation and further insights are then achieved by screening for and characterising responsible enzymes.

4. “the involvement of the Pkh1/2/3 kinases in Pil1 phosphorylation”
This misses the point of our first two figures, which we now explain more clearly in the first paragraph of the Results section (**Page 2; line 53**). Before our manuscript, it was assumed that all three Pkh-kinases localised to eisosomes and were equally responsible for Pil1 phosphorylation. Only Pkh1 and Pkh2 have ever been localised so far, using reasonable tools at the time - that unfortunately required glucose starvation media conditions to (highly) overexpress the GFP tagged kinases. We clarify for the first time that at steady state, only Pkh2 localises to eisosomes. Previous work in cerevisiae has also never tested the role of *pkh1Δ* or *pkh3Δ* cells alone (only using *pkh1 pkh2* double mutants). We show at a functional level, for the first time, Pkh1 and Pkh3 have very little to do with Pil1 phosphorylation under normal growth conditions.

Specific comments:
-Loss of Sit4 has a strong effect on Pil1 phosphorylation. This is not further investigated because of the lack of Sit4 localization to the periphery. I am not convinced that the localization of the tested kinases is that meaningful since Pil1 is dynamically cycling on and off eisosomes and therefore some of the regulation might occur in the cytoplasm.

We now include several sentences and citations to discuss our discovery that deletion of both Sit4 and Yvh1 results in defective Pil1 dephosphorylation (**Page 5; line 53**). However, we consider functional characterisation of either enzyme, or testing whether they act directly or indirectly on Pil1, far beyond the scope of this manuscript (which already contains ~50 main figure subpanels + supplements).

Although Glc7, Sit4 and Yvh1 were all in the top seven qualitative (Figure provided for reviewer has been removed – **Figure 4B**) and top three quantitative (Figure provided for reviewer has been removed – **Figures 5A, 5B**) phosphatase screen hits, we chose to focus on Glc7 for this paper because it was the only of these enzymes with peripheral localisation (Figure provided for reviewer has been removed – **Figure 5C, S4**), that colocalised with eisosomes (Figure provided for reviewer has been removed - **Figures 6G, S5D**), and was linked conceptually to glucose starvation in the literature.

-The eisosome defect caused by the 8D or 8A mutant versions of Pil1 is severe and therefore it is not surprising that these mutants show similar starvation recovery defects as pil1 deletions.

We agree that it is perhaps not surprising that phospho-locked 8A and 8D mutants result in severe mis-localization and starvation recovery phenotypes. However, we also felt it was very important to perform these experiments and to document the results if only to contextualise our more nuanced experiments with *glc7* mutants.

We have moved the recovery assay data for all these different experiments to a new expanded Figure provided for reviewer has been removed – **Figure 8**, to help make these connections and comparisons easier. We also include more new data about the localisation of the phospho-mutants mentioned in cells with eisosomes marked with mCherry tagged Can1 (Figure provided for reviewer has been removed – **Figure 7F**).

© 2023. Published by The Company of Biologists under the terms of the Creative Commons Attribution License (https://creativecommons.org/licenses/by/4.0/).

-The manuscript states that transporters such as Mup1 are moving to eisosomes during glucose starvation. This is somewhat misleading since a large portion of nutrient transporters are endocytosed and degraded during starvation. Only a subset of the transporters remain at the surface and this subset localizes to eisosomes.

Thank you for pointing out this misleading statement. We now clarify (**Page 3; line 32**) between endocytosed and eisosome retained populations, which we have previously characterised and quantified (Laidlaw *et al*., J. Cell Sci. 2021).

Second decision letter

MS ID#: JOCES/2022/260505

MS TITLE: The phosphatase Glc7 controls eisosomal response to starvation via posttranslational modification of Pil1

AUTHORS: Katherine M Paine, Kamilla ME Laidlaw, Gareth J.O Evans, and Chris MacDonald
ARTICLE TYPE: Research Article

I am happy to tell you that your manuscript has been accepted for publication in Journal of Cell Science, pending standard ethics checks. I leave it up to you if you feel that the addition of the paper (DOI: 10.7554/eLife.58246) suggested by reviewer 1 should be added before the finalversion of the paper appears.

Reviewer 1

Advance summary and potential significance to field

The author show that the phosphatase Glc7 controls eisosomal response to starvation via posttranslational modification of Pil1. This process helps to adapt nutrient acquisition and thus provides a better understanding of how cells regulate the transport nutrients across their membranes.

Comments for the author

The authors have addressed my major points in the revised version of their manuscript. The new data supports their original conclusion and bolsters their attractive model on how the regulation of eisosomes ('caveolae like' PM subdomains) modulates nutrient transporter retention at the PM to allow efficient recovery and growth after starvation. I only have a minor optional request / suggestion: In the introduction, where the authors describe how cells coordinate selective nutrient transporter trafficking in response to nutritional cues, I think it might be worthwhile to cite Ivashov et al. (DOI: 10.7554/eLife.58246). This paper helps to explain how different metabolic cues activate distinct α-arrestin-Rsp5-complexes to control context specific nutrient transporter ubiquitination and endocytosis for the selective remodeling of the nutrient transporter landscape. I would like point out that this request is optional, and should only be considered if the authors think along the same lines.

Reviewer 2

Advance summary and potential significance to field

The present study identifies the kinase Pkh2 and the phosphatase Glc7 as major modifiers of the eisosomal protein Pil1. The nutrient status of the cell affects Pil1 phosphorylation, which in turn

© 2023. Published by The Company of Biologists under the terms of the Creative Commons Attribution License (https://creativecommons.org/licenses/by/4.0/).

seems to affect the localization of nutrient transporters to eisosomes. The data suggest that the eisosomal storage of transporters observed during glucose starvation is dependent on the Glc7-mediated dephosphorylation of Pil1. Loss of GLC7 function results in a delayed recovery from starvation, possibly because of lack of nutrient transporters at the cell surface.

Comments for the author

The revisions addressed my main concerns and therefore I am supporting the publication of this article.

© 2023. Published by The Company of Biologists under the terms of the Creative Commons Attribution License (https://creativecommons.org/licenses/by/4.0/).
